# Epigenetic Age Acceleration of Stomach Adenocarcinoma Associated With Tumor Stemness Features, Immunoactivation, and Favorable Prognosis

**DOI:** 10.3389/fgene.2021.563051

**Published:** 2021-03-18

**Authors:** Chunhong Hong, Shaohua Yang, Qiaojin Wang, Shiqiang Zhang, Wenhui Wu, Jinyao Chen, Danhui Zhong, Mingzhe Li, Liang Li, Jianfeng Li, Hong Yu, Hong Chen, Qianlin Zeng, Changhua Zhang

**Affiliations:** ^1^Center of Digestive Disease, The Seventh Affiliated Hospital, Sun Yat-sen University, Shenzhen, China; ^2^Department of Surgical Intensive Care Unit, The First Affiliated Hospital, Sun Yat-sen University, Guangzhou, China; ^3^Department of Urology, The Seventh Affiliated Hospital, Sun Yat-sen University, Shenzhen, China; ^4^Department of Physiotherapy, The University of Hongkong-Shenzhen Hospital, Shenzhen, China

**Keywords:** DNA methylation age, stomach adenocarcinoma, prognosis, immunoactivation, tumor stemness

## Abstract

**Background:** Abnormal DNA methylation (DNAm) age has been assumed to be an indicator for canceration and all-cause mortality. However, associations between DNAm age and molecular features of stomach adenocarcinoma (STAD), and its prognosis have not been systematically studied.

**Method:** We calculated the DNAm age of 591 STAD samples and 115 normal stomach samples from The Cancer Genome Atlas (TCGA) and gene expression omnibus (GEO) database using the Horvath’s clock model. Meanwhile, we utilized survival analysis to evaluate the prognostic value of DNAm age and epigenetic age acceleration shift. In addition, we performed weighted gene co-expression network analysis (WGCNA) to identify DNAm age-associated gene modules and pathways. Finally, the association between DNAm age and molecular features was performed by correlation analysis.

**Results:** DNA methylation age was significantly correlated with chronological age in normal gastric tissues (*r* = 0.85, *p* < 0.0001), but it was not associated with chronological age in STAD samples (*r* = 0.060, *p* = 0.2369). Compared with tumor adjacent normal tissue, the DNAm age of STAD tissues was significantly decreased. Meanwhile, chronological age in STAD samples was higher than its DNAm age. Both DNAm age and epigenetic acceleration shift were associated with the prognosis of STAD patients. By using correlation analysis, we also found that DNAm age was associated with immunoactivation and stemness in STAD samples.

**Conclusion:** In summary, epigenetic age acceleration of STAD was associated with tumor stemness, immunoactivation, and favorable prognosis.

## Introduction

Gastric cancer (GC) is one of the most common gastrointestinal tract malignancy and it is also the leading causes of cancer-related mortality worldwide (Van Cutsem et al., 2016; Siegel et al., 2018; Sitarz et al., 2018). Stomach adenocarcinoma (STAD) comprises 95% of gastric cancer, which primary arises from gastric epithelium including gastric mucosa and superficial glands of the epithelium (Schwartz, 1996; Dicken et al., 2005). Immune microenvironment in STAD has been increasingly studied in recent years (Couderc et al., 1987; Leone et al., 2017; Patil et al., 2018; Jiang et al., 2019; Li et al., 2019; Zeng et al., 2019). In addition, immune cell infiltration, such as T helper (Th), Th2, and mast cells, has been reportedly related to the prognosis of STAD patients (Wang et al., 2020).

Epigenetic clock or DNA methylation (DNAm) age was found to be highly correlated with chronological age (CA) in most tissues and cell types in human (Horvath, 2013, 2015). Generally speaking, DNAm age in different tissues and cell types shared the following characteristics: first, the DNAm age of embryonic and induced pluripotent stem (iPS) cells was close to zero; second, DNAm age was correlated with the number of cell passages; and third, DNAm age caused a highly heritable measure of chronological age acceleration (Horvath, 2013). Vertical shift between DNAm age and CA, or epigenetic acceleration shift, has been reported to be closely associated with many cancer types, including cervical, breast, and lung cancer (Levine et al., 2015; Ambatipudi et al., 2017; Lu et al., 2020). A recent study found that DNAm age was significantly associated with CA in normal cervical tissues and cervical squamous cell carcinoma (CSCC) samples. In addition, DNAm age acceleration of CSCC leads to immunoactivation, human papillomavirus 16/18 expression, and better prognosis (Lu et al., 2020). Interestingly, prognostic effects of DNAm age acceleration in breast carcinoma show opposite conclusions in two studies (Ren et al., 2018; Kresovich et al., 2019). One of them stated that DNAm age acceleration was significantly associated with oncogenesis in breast cancer (Kresovich et al., 2019). However, the other argued that DNAm age acceleration was significantly associated with better prognosis after adjusting for clinicopathological variables, such as estrogen receptor status and cancer stage (Ren et al., 2018). The possible reasons for the discrepancies between them are as follows: (i) the clinical samples used in the two studies were different. In Kresovich et al.’s research, DNAm age for each breast cancer patients were calculated based on blood samples. On the other hand, DNAm age for each breast cancer patients in Ren et al.’s paper was calculated based on cancer tissue samples; (ii) the outcome indicators of the two studies were different. In Kresovich et al.’s study, the outcome indicator was the occurrence of breast cancer during the 5-year observation period. On the other hand, the outcome indicators of Ren et al.’s study were the prognosis and recurrence of breast cancer patients; and (iii) the variables included in the two studies are different, which may have an impact on the model. Taken together, these studies indicated a new perspective on the pathogenesis and prognostic biomarker of cancer.

Although epigenetic age shift in some studies has been assumed to be an indicator for canceration and all-cause mortality, the DNAm age in STAD and its clinical value remains poorly understood. In this study, we focused on STAD and systematically analyzed associations of epigenetic age with molecular characteristics and clinical outcomes in STAD patients. Briefly, the correlation between DNAm age and CA in STAD samples was impaired, and epigenetic acceleration shift was associated with a favorable prognosis in STAD patients. In addition, DNAm age was associated with immunoactivation and tumor stemness features in STAD samples.

## Materials and Methods

### Data Acquisition From the TCGA and GEO Database

**Figure 1:**
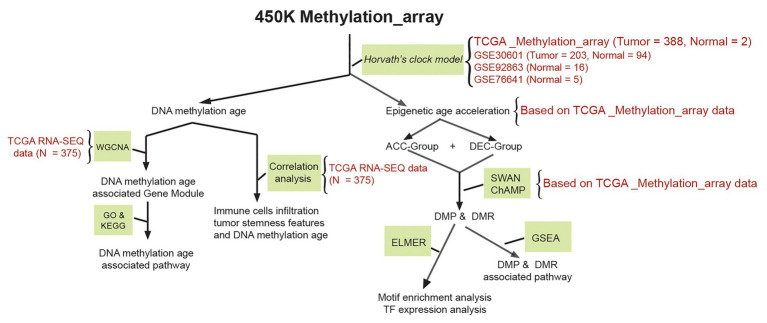
Schematic of the study method. Methylation array data of stomach adenocarcinoma (STAD) and normal stomach were downloaded from The Cancer Genome Atlas (TCGA) and gene expression omnibus (GEO) database. TCGA_Methylation_array, GSE30601, GSE92863, and GSE76641 datasets were used to calculate DNAm age based on a Horvath’s clock model; TCGA RNA-SEQ data were used to identify DNAm age related gene module based on WGCNA; relationship between DNAm age and immune cell infiltration profiles of STAD patients calculated by TCGA RNA-SEQ data were evaluated by correlation analysis; epigenetic age acceleration of STAD patients, DMP, and DMR were calculated based on TCGA_Methylation_array data. STAD, stomach adenocarcinoma; TCGA, The Cancer Genome Atlas; GEO, the gene expression omnibus; DMP and DMR, differential methylated probes and differential methylated regions; ACC, DNAm age accelerated group; DEC, DNAm age decelerated group; GSEA, gene set enrichment analysis; ELMER, enhancer linking by methylation/expression relationships.

Both 27 K and 450 K array data from Illumina Infinium platform can be used to calculate epigenetic age based on a Horvath’s clock model (Horvath, 2013). We therefore downloaded human-methylation 450 K array data of STAD patients including 388 STAD and two tumor adjacent normal tissues from the Santa Cruz Xena Public Data Hubs of the University of California.^1^ Corresponding clinical information of STAD samples was obtained from Cruz Xena Public Data Hubs and cBioPortal website.^2^ Due to the limitation samples size of normal gastric tissue, another 115 normal gastric samples were obtained from gene expression omnibus (GEO) online database.^3^ Both GSE92863 (Yamashita et al., 2019; 16 normal gastric samples) and GSE76641 (Roost et al., 2017; five normal gastric samples) datasets based on Illumina human-methylation 450 K array data which contains age information. However, GSE30601 (Zouridis et al., 2012) dataset which contained 203 gastric cancer and 94 tumor adjacent normal tissues based on Illumina human-methylation 27 K array data without detailed age information. We, therefore, used GSE30601 dataset to compare the DNAm age difference between STAD and its corresponding paired tumor adjacent normal samples. The mRNA expression profile of 375 STAD and 32 tumor adjacent normal tissues were downloaded from The Cancer Genome Atlas (TCGA) database (https://portal.gdc.cancer.gov/repository). The RNA-SEQ data were quantified as raw read count for differential expression gene (DEG) analysis. RNA-SEQ counts data of STAD samples were transferred into transcripts per kilobase million (TPM) values, and were then used to calculate immune cells infiltration profiles and tumor stemness features. Figure 1 illustrates the protocol of bioinformatics analyses.

### DNA Methylation Age and Epigenetic Age Shift

DNA methylation age was calculated by the Horvath’s clock model based on methylation *β* value matrix. The *β* values for each site were calculated by the following equation: *β* = Mean_methylated (*M*)/[Mean_methylated (*M*) + Unmethylated (*U*)]. Function to calculate DNAm age in the Horvath’s clock model was obtained from Horvath’s article (Horvath, 2013) and performed in R software (version 3.5.1). Mathematics of DNAm age calculation function in the Horvath’s clock model is briefly described as follows: *DNAmage = inverse.F* (*b_0_ + b_1_CpG_1_ + … + b_353_CpG_353_*). *F* is a function used for age transformation and 353 *b_i_* coefficients were obtained from elastic net regression results in a linear regression model. Vertical shift (i.e., acceleration) of epigenetic age was calculated by the formula *E-value = DNAmage_n_ − CA_n_* where *CA_n_* is the chronological age of each STAD sample. We therefore divided the STAD samples into epigenetic age accelerated (DNAmAge-ACC, *E-value* > 0) and epigenetic age decelerated group (DNAmAge-DEC, *E-value* < 0).

### Differential Methylated Probes and Motif Enrichment Analysis

Stomach adenocarcinoma methylation array data obtained from Cruz Xena Public Data Hubs was primarily presented as a *β* value matrix. Firstly, we filtered and normalized this *β* value matrix by using *SWAN* method embedded in ChAMP package (version 2.13.5). Then, differential methylated probes (DMPs) between DNAmAge-ACC and DNAmAge-DEC group were identified by the *champ.DMP ()* function with the parameters adjusted *p* < 0.05 and delta *β* > 0.1. In addition, gene set enrichment analysis (GSEA) based on DMPs was performed by champ.GSEA *()* function in ChAMP package. ChAMP used pathway information downloaded from MSigDB. Then Fisher Exact Test will be used to calculate the enrichment status of each pathway. After gene enrichment analysis, champ.GSEA *()* function would automatically return pathways with value of *p* smaller than adjPval cutoff. Finally, *get.enriched.motif ()* in Enhancer Linking by Methylation/Expression Relationships (ELMER) package was used to find motif occurrences in differential methylated positions. *Fisher’s* and *Benjamini-Hochberg* test were performed to correct the background of all DMPs regions. According to the default parameters of the *get.enriched.motif ()*, the *motif occurred frequency* > 10 times and 95% confidence interval of the *odds ratio value* > 1.1 was considered as significant motif.

### DEG and Function Annotation Analysis

Differential expression genes between STAD and tumor adjacent normal tissues obtained from TCGA online database were identified by edgeR packages in R software. In addition, we also used edgeR function to screen the DEGs DNAmAge-ACC and DNAmAge-DEC group. In this section, an adjusted *p* < 0.05 and *fold change* > 2.0 were considered as statistically significant. To investigate the potential mechanism of epigenetic age shift in canceration and prognosis of STAD, enrichment function annotation analysis was performed based on DEGs by using the org.Hs.eg.db and ClusterProfiler package (Yu et al., 2012) in R software and an *adjusted p* < 0.05 was considered as statistically significant. In addition, single gene GSEA was performed based on the median transcripts per million (TPM) value of each TF, STAD samples were divided into high and low expression groups. Then, GSEA analysis was performed to identify pathway enriched between TF high and low expression groups. In addition, permutation test was performed to calculate value of *p* in single gene GSEA analysis (Yu et al., 2012). Reference gene-set (c2.cp.kegg.v6.2.symbols.gmt) was downloaded from MSigDB gene set hub^4^ and a value of *p* less than 0.01 was considered as statistically significant.

### Weight Gene Co-expression Network Analysis

In order to further study the relationship between gene transcripts and DNAm age in STAD, we, therefore, further used gene co-expression network analysis to identify DNAm age-associated gene modules and pathways. Weight gene co-expression network analysis (WGCNA) was performed through the WGCNA package in R software as previously described (Zheng et al., 2019). Briefly, a pairwise coefficient matrix was first calculated by using correlation analysis (*Pearson*) and further converted into an adjacency matrix (weighted) based on a soft threshold. Then, a topological overlap matrix (TOM) was computed using *blockwiseModules ()* function in WGCNA package based on the previous adjacency matrix. Hierarchical clustering and dynamic tree cut algorithm in WGCNA package were used to identify gene modules. In addition, module eigengene (ME) was considered as a summary value of gene expression profiles for the given gene module. Finally, correlation analysis (*Pearson*) was performed to calculate the correlation coefficient between ME and external DNAm age value.

### Immune Cells Infiltration Profile and Tumor Stemness Features Analysis

We performed xCell algorithm-based on single-sample gene set enrichment analysis (ssGSEA) in R software to compute the enrichment score of 64 immune and stromal cell types in STAD samples (Aran et al., 2017). Briefly, enrichment score of 64 cell types are estimated by 489 gene signatures, and the primary enrichment score of all gene signatures corresponding to target cell type are averaged. Then, a cell matrix was generated including 34 types of immune cells and 30 types of other cells of each STAD sample. Immune cells can be further classified into nine categories, including CD8 T-cell subpopulations, CD4 T-cell subpopulations, gamma delta T cells (Tgd cells), B-cell subpopulations, monocyte/macrophage subpopulations, dendritic cell (DC) subpopulations, granulocyte subpopulations, nature killer (NK) cells, and NKT cells subpopulations. In addition, three summary score including immune-score, stroma-score, and microenvironment-score were also calculated for further analyses. One-class logistic regression (OCLR) machine-learning method based on pluripotent stem cell samples dataset was used to calculate the mRNA stemness index (mRNAsi) in STAD samples (Salomonis et al., 2016; Daily et al., 2017; Malta et al., 2018). The detail method to compute mRNAsi has been well noted in Malta’s paper (Malta et al., 2018) and is available on https://bioinformaticsfmrp.github.io/PanCanStem_Web/. According to OCLR algorithm, the mRNAsi of each sample was mapped to range 0–1 *via* utilizing a linear transformation model that subtracted the minimum value and divided by the maximum value.

### Statistical Analysis

In this study, continuous variables were described as mean and *SD*, or median and quartiles (*Q*), depending on the value distribution of each variable which was tested by the *Shapiro-Wilk* test. Categorical code variables were reported as frequencies and proportions. The statistical analysis used to compare the difference between DNAmAge-ACC and DNAmAge-DEC groups included *two independent samples t-test* and *paired samples t-test* for mean values, *Mann-Whitney U-test* for median values, and *Fisher’s exact test* for frequencies and proportions variables. The correlation between two continuous variables was tested by *Pearson (R)* and *Spearman (Rho)* coefficients based on data distribution. In addition, the difference between two correlation coefficients was tested by *Z-statistic*. The prognostic value of DNAm age (or epigenetic age acceleration shift), transcription factors (TFs), immune cells, and mRNAsi were evaluated by *Kaplan-Meier* curve and *univariate Cox regression model* in R software (*survival* package). In this study, all statistical tests were performed in *R* software (*version 3.5.1*; Microsoft, Redmond, WA, United States) and a *p* < 0.05 (*two-tailed*) was served as a statistically significant level.

## Results

### Correlation Between Epigenetic and Chronological Age in Stomach Adenocarcinoma and Para-Cancer Tissues

**Figure 2:**
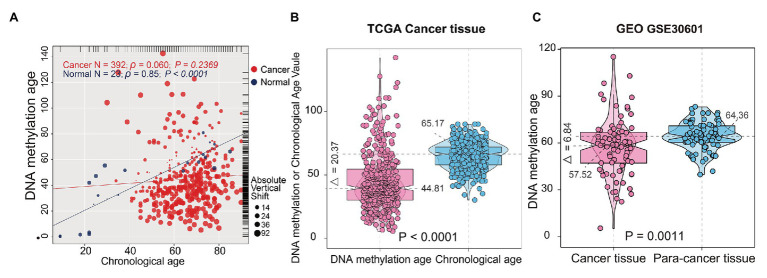
Correlations between DNAm age and chronological age (CA) in stomach tissues. **(A)** Correlations between DNAm age and CA in normal gastric and STAD samples; **(B)** DNAm age was significantly lower than CA in TCGA STAD samples. **(C)** Tumor adjacent normal tissues presented with significantly higher DNAm age than cancer tissues. The test for association between gastric samples used Pearson’s correlation coefficient. Two-tailed statistical *p* values were calculated by a paired samples *t*-test. STAD, stomach adenocarcinoma; TCGA, The Cancer Genome Atlas.

DNA methylation age was significantly correlated with CA in normal gastric tissues (*r* = 0.85, *p* < 0.0001, *n* = 23), but it was not associated with CA in STAD samples (*r* = 0.060, *p* = 0.2369, *n* = 388; Figure 2A), which indicated that the epigenetic pattern observed in normal gastric samples was disrupted in STAD samples. In normal gastric tissues, there was no difference between DNAm age and CA (*p* = 0.2826; paired samples *t*-test; Supplementary Figure 1); Meanwhile, CA in STAD samples were higher than DNAm age (*p* < 0.0001; paired samples *t*-test; Figure 2B). In addition, compared with para-cancer tissues, the DNAm age in STAD tissues was significantly decreased (*p* = 0.0011; independent samples *t*-test; Figure 2C). These results indicated that DNAm age deceleration may play an important role in tumorigenesis of STAD.

### Association Between Epigenetic Age Shift and Prognosis

**Figure 3:**
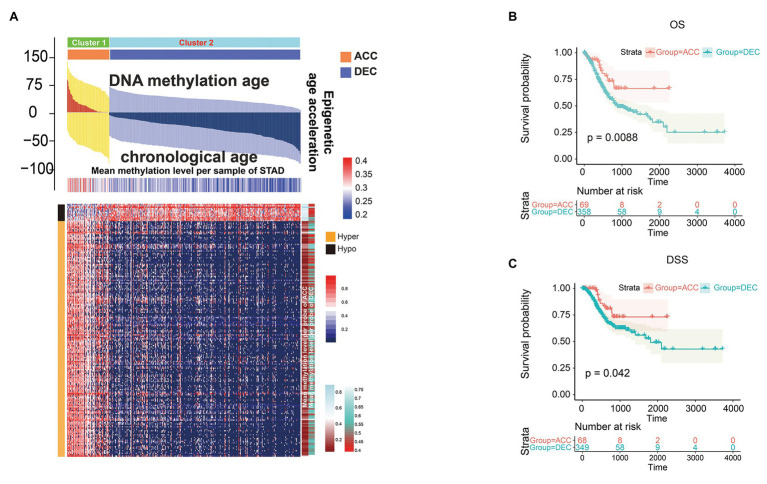
Prognostic value of epigenetic age acceleration in STAD patients. Heatmap showing differential DNAm pattern in CpG islands between DNAmAge-ACC and DNAmAge-DEC group **(A)**. Patients belonging to DNAmAge-ACC showed significantly favorable prognosis in terms of overall survival (OS; **B**) and disease-specific survival (DSS; **C**). The association between DNAm age shift value and the prognosis of STAD including OS **(B)** and DSS **(C)** was presented with cubic spline graphs of the hazard ratio (solid red line) and 95% confidence interval (dotted black line). OS, overall survival; DSS, disease specific survival. ACC, accelerated; DEC, decelerated; STAD, stomach adenocarcinoma.

We then studied whether DNAm age was associated with the prognosis of STAD patients and found that higher DNAm age was associated with better overall survival (*OS*; *p* = 0.038) according to *Kaplan-Meier* survival log-rank test (Supplementary Figure 1B). In addition, higher *DNAm age* acceleration value was associated with better disease-specific survival (*DSS*) event when using median and quartile acceleration value, respectively, (*p* = 0.031 and *p* = 0.049; Supplementary Figures 1G,K). To further study the relationship between epigenetic clock shift and STAD patients’ prognosis, we divided STAD patients into DNAmAge-ACC and DNAmAge-DEC groups according to *E-value* > 0 or *E-value* < 0. In this study, 318 STAD patients were identified as DNAmAge-DEC group, while only 70 patients were identified as DNAmAge-ACC group (Figure 3A; Table 1). In addition, most STAD samples in DNAmAge-ACC group showed higher mean methylation level, while most samples in DNAmAge-DEC group showed lower mean methylation level (Figure 3A). These results suggested that STAD patients in TCGA cohort include two clusters: DNAmAge-ACC cluster (low proportion; Figure 3A) and DNAmAge-DEC (high proportion; Figure 3A). We then studied whether this classification pattern was related to STAD patients’ prognosis, and found that DNAmAge-ACC was associated with favorable OS and DSS (*p* = 0.0088 and *p* = 0.042, respectively; Figures 3B,C).

**Table 1:** Demographic and clinicopathological characteristic between DNA methylation (DNAm) age accelerated group (ACC) and DNAm age decelerated group (DEC).

Characteristics	Total (%)	Epigenetic age (0-year cut-off)		*p*^a^
		DNAmAge-ACC (*N* = 70)	DNAmAge-DEC (*N* = 318)	
Age (years)^b^	388 (100)	61.4 ± 12.2	66.0 ± 10.2	0.004
Fraction_Genome_Altered^b^	353 (91)	0.3 ± 0.2	0.2 ± 0.2	0.342
Mutation_Count^b^	311 (80)	371.2 ± 650.4	399.5 ± 620.4	0.741
Stage				0.3632
I+II	174 (44.8)	32 (45.7)	142 (44.7)	
III+IV	203 (52.3)	38 (54.3)	165 (51.9)	
Missing	11 (2.8)	0 (0)	11 (3.5)	
Race				0.3554
White	252 (64.9)	47 (67.1)	205 (52.8)	
Others	102 (26.3)	20 (28.6)	82 (21.1)	
Missing	34 (8.8)	3 (4.3)	31 (7.9)	
Sex				0.7831
Male	244 (62.9)	42 (60)	202 (63.5)	
Female	111 (28.6)	21 (30)	90 (28.3)	
Missing	33 (8.5)	7 (10)	26 (8.2)	
Person_Neoplasm_Status				0.6043
With tumor	76 (19.6)	11 (15.7)	65 (20.4)	
Tumor free	226 (58.2)	41 (58.6)	185 (58.2)	
Missing	86 (22.2)	18 (25.7)	68 (21.4)	
Overall Survival				0.0580
Live	237 (61.1)	50 (71.4)	187 (58.8)	
Death	151 (38.9)	20 (28.6)	131 (41.2)	
Disease-specific survival				0.268
Live	100 (25.8)	13 (18.6)	87 (27.4)	
Death	279 (87.7)	56 (80.0)	223 (70.12)	
Missing	9 (2.8)	1 (1.4)	8 (2.5)	

a Fisher’s exact test for categorical data and a two-sample Mann-Whitney test for continuous data.

b Continuous values are represented with mean ± SD.

### Molecular Features Between DNAmAge-ACC and DNAmAge-DEC Group

**Figure 4:**
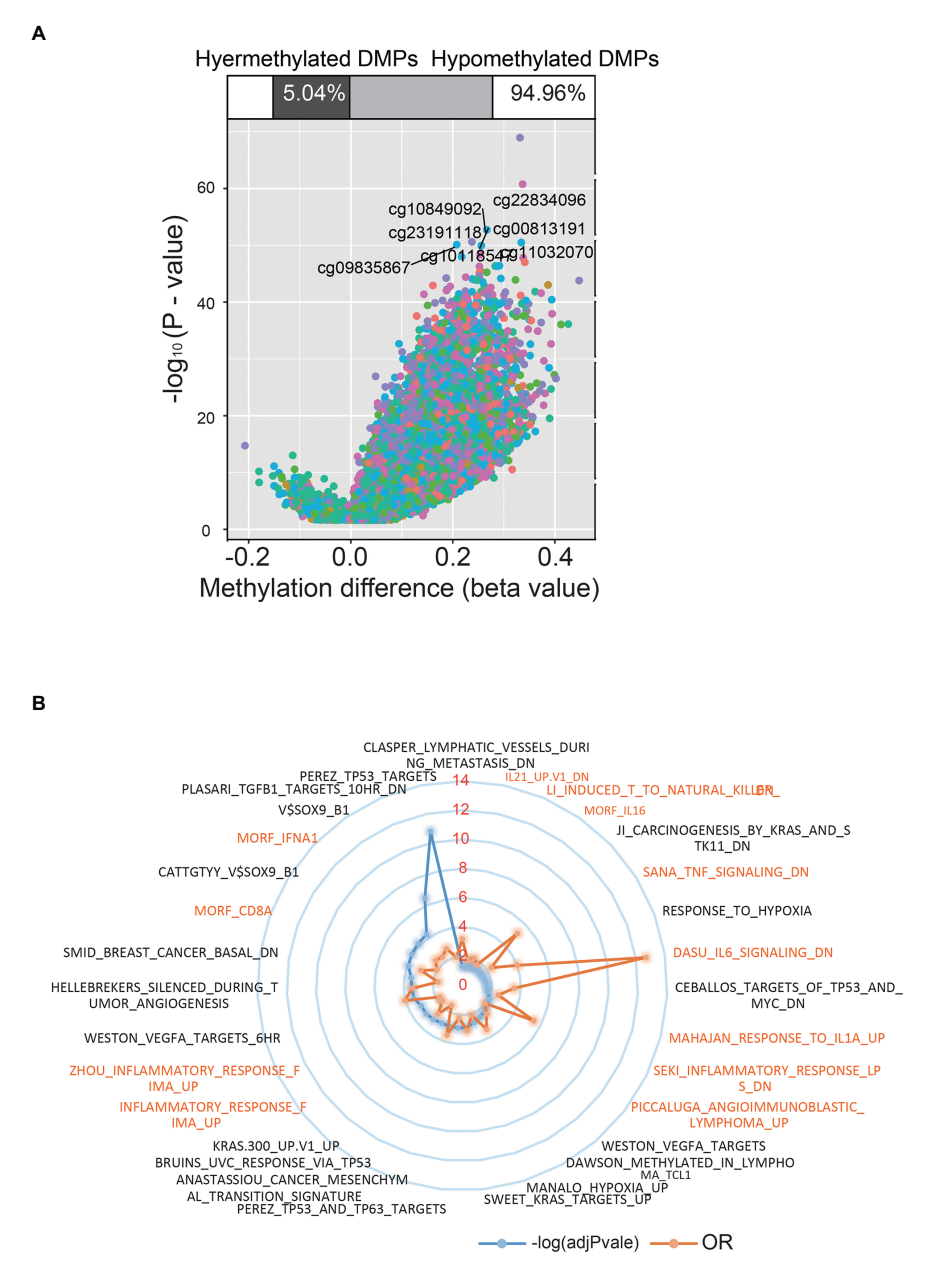
Differential methylation sites between DNAmAge-ACC and DNAmAge-DEC group. Using ChAMP, we identified 77,186 differential methylation probes in samples of 70 DNAmAge-ACC and 318 DNAmAge-DEC samples **(A)**. Gene set enrichment analysis (GSEA) identified immune-related signaling pathways **(B)**.

Except for age, the distribution of patients’ clinical stage, fraction genome altered, mutation count, race, sex, neoplasm status, and survival event showed no significant difference between DNAmAge-DEC and DNAmAge-ACC groups (Table 1), which indicated that the demographical and clinicopathological characteristics of DNAmAge-DEC and DNAmAge-ACC groups were consistent and reasonable for use in addition to molecular analyses. We then used *champ.DMP ()* and *champ.DMR ()* function in R software to screen the DMPs and DMRs between DNAmAge-DEC and DNAmAge-ACC groups. In this study, we identified 77,186 DMPs (*p* < 0.05 and |delta *β*| > 0.1) and 2008 DMRs (*p* < 0.05) in the samples including 70 DNAmAge-ACC and 318 DNAmAge-DEC samples (Figure 4A). Meanwhile, genome location analysis based on human methylation 450 bead-chip annotation platform showed that the gene body and intergenic region shared the highest proportion of DMPs, 31.6 and 26.3%, respectively (Figure 4A). Most DMPs (94.96%) between DNAmAge-ACC and DNAmAge-DEC group were identified as hypermethylation DMPs which are highly consistent with the Horvath’s clock model and clusters pattern (Figure 4A). We then performed GSEA to study the biological functions of DMPs in STAD patients and found that those CpG sites were significantly enriched in immune, tumor protein p53 (TP53), vascular endothelial growth factor A (VEGFA), and hypoxia-associated pathways (Figure 4B). To further study the effect of DMPs on mRNA transcriptional phenotype, we then performed DEGs analysis between DNAmAge-ACC and DNAmAge-DEC groups and found that those DEGs were also significantly enriched in immune-associated pathways (Supplementary Figures 2A–D). In addition, 93.1% (1,659/1782) of the genes in DEGs were low expression (fold change < 0; Supplementary Figure 2E), which is highly consistent with DMPs between DNAmAge-ACC and DNAmAge-DEC groups.

### Enriched Motifs of Differentially Methylated Sites

**Figure 5:**
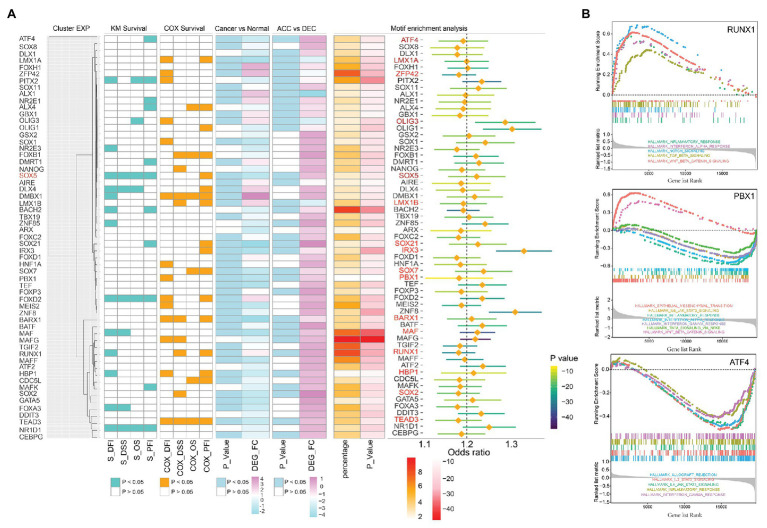
The expression profiles and prognosis effect of enriched transcription factors (TFs) in TCGA STAD cohort. **(A)** Prognosis-associated TFs (including ATF4, LMX1A, ZFP42, OLIG3, SOX5, LMX1B, SOX21, IRX3, SOX7, PBX1, BARX1, MAF, RUX1, HBP1, SOX2, and TEAD3) and differential expressed genes were showed on the left side of the figure; enriched TFs and related odds ratio values were showed on the right side of the figure. **(B)** GSEA identified TFs including RUNX1, PBX1, and ATF4 were significantly enriched in immune-related pathways. The genes were ranked by log_2_ fold change. DEG, differential expression genes; OS, overall survival; DSS, disease specific survival; PFI, progression free interval event; DFI, disease free interval event; S, results of Kaplan-Meier survival analysis; COX, results of univariate Cox regression analysis; ACC, DNAm age accelerated group; DEC, DNAm age decelerated group; STAD, stomach adenocarcinoma; TCGA, The Cancer Genome Atlas.

To further study the regulation mechanism of epigenetic age shift in STAD, we performed motif enrichment analysis based on ELMER package and identified 366 significantly enriched motifs in different methylation sites (*p* < 0.05 and odds ratio value > 1.1; Supplementary Table S1). We next sought to determine the expression profiles and prognosis effect of enriched motifs-associated TFs in TCGA STAD cohort. The expression level of the candidate genes was used to hierarchical clustering analysis. As showed in Figure 5A, candidate genes were divided into three clusters. In addition, enriched motifs and related TFs in three independent cluster such as activating transcription factor 4 (ATF4), LIM homeobox transcription factor 1 alpha (LMX1A), ZFP42 zinc finger protein (ZFP42), oligodendrocyte transcription factor 3 (OLIG3), SRY-Box transcription factor 5 (SOX5), LIM homeobox transcription factor 1 beta (LMX1B), SRY-Box transcription factor 21 (SOX21), iroquois homeobox 3 (IRX3), SRY-Box transcription factor 7 (SOX7), PBX homeobox 1 (PBX1), BARX homeobox 1 (BARX1), MAF BZIP transcription factor (MAF), RUNX family transcription factor 1 (RUNX1), HMG-Box transcription factor 1 (HBP1), SRY-Box transcription factor 2 (SOX2), and TEA domain transcription factor 3 (TEAD3) were related to the prognosis of STAD patients. These prognosis-associated TFs were also identified as DEGs between DNAmAge-ACC and DNAmAge-DEC groups as well as between cancer and tumor adjacent normal tissues (Figure 5A). In addition, some of these TFs such as ATF4, PBX1, and RUNX1 were significantly enriched in immune, epithelial mesenchymal transition (*EMT*), Wingless-type (*Wnt*), and Notch signaling associated pathway (Figure 5B).

### Epigenetic Age Associated Gene Modules in TCGA STAD Cohort

**Figure 6:**
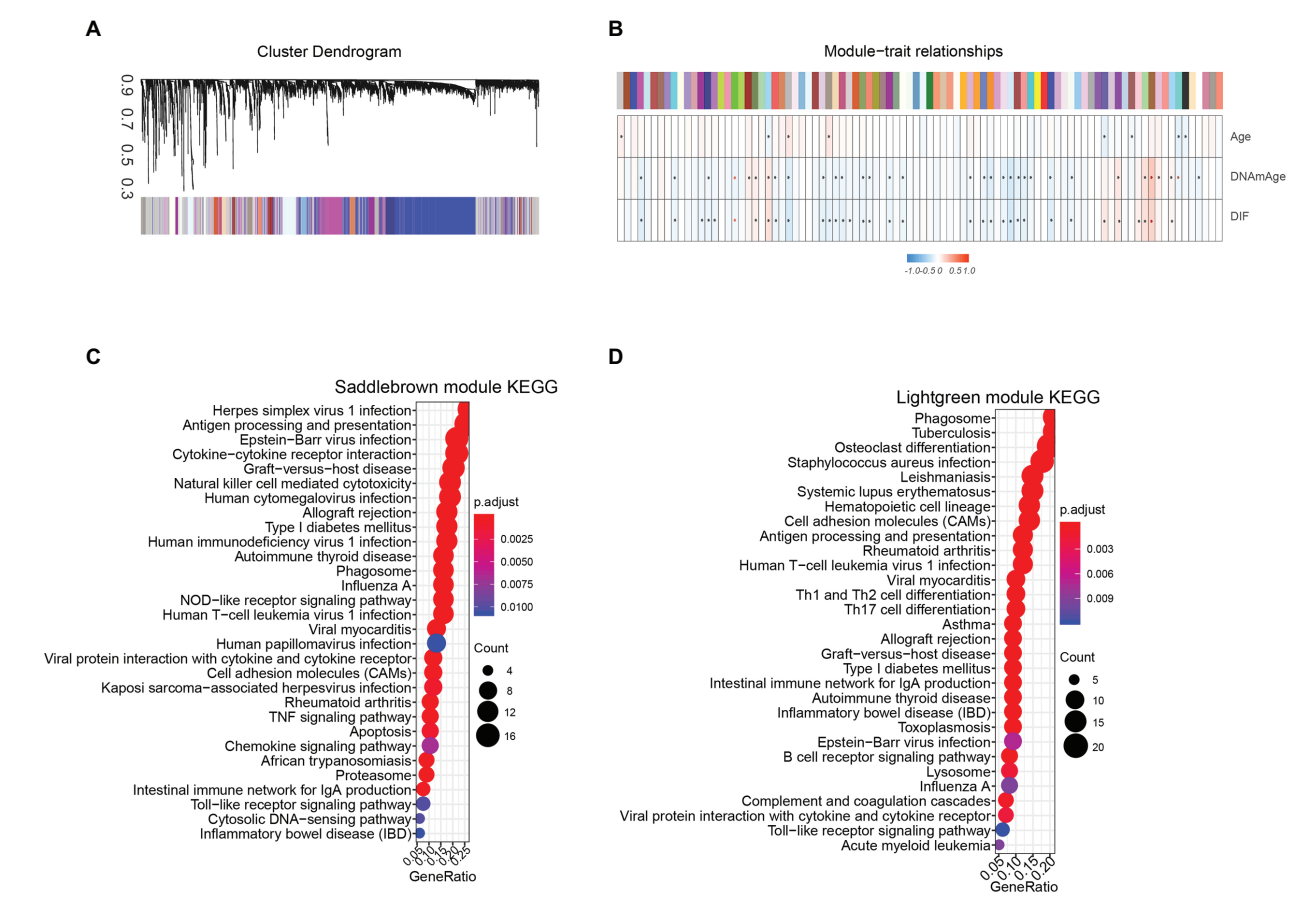
Epigenetic age associated gene modules in TCGA STAD cohort. **(A)** Using weighted gene co-expression network analysis, we identified 90 co-expressed gene modules in TCGA STAD cohort. Average linkage hierarchical clustering dendogram of the genes. Input was the topological overlap-based dissimilarity. Modules, which are designated by color code, are the branches of the clustering tree. **(B)** Eight gene modules were positively correlated with DNAm age in STAD patients. Twenty-one gene modules were negatively correlated with DNAm age in STAD patients. Color represents independent gene module, Correlation of module eigengenes to clinical and pathological traits. Each column corresponds to a module eigengene, and the row represents the DNAmage variables. ^*^represent *p* < 0.05, DIF, DNAmage shift value. Genes in top two DNAm-age-associated gene modules, including Saddlebrown **(C)** and Lightgreen **(D)** modules, were significantly enriched in immune-related pathways. STAD, stomach adenocarcinoma; TCGA, The Cancer Genome Atlas.

Using WGCNA algorithm, we identified 90 co-expressed gene modules in TCGA STAD cohort (Figure 6A) based on a threshold value of 6 (Supplementary Figure 3). We then performed *Pearson* correlation analysis to calculate the coefficients between each genes module and DNAm age. Eight gene modules were positively correlated with the DNAm age of STAD patients (*p* < 0.05; Figure 6B). On the other hand, 21 gene modules were negatively correlated with DNAm age in STAD patients (*p* < 0.05; Figure 6B). In addition, we performed gene ontology (GO) and Kyoto Encyclopedia of Genes and Genomes (KEGG) analysis to study the biological functions of genes in top two DNAm-age associated gene modules (saddlebrown and lightgreen) and found that these genes were significantly enriched in immune related pathways including antigen processing and presentation, Th1 and Th2 cell differentiation, Th17 cell differentiation, natural killer cell mediated cytotoxicity, and toll-like receptor signaling pathways (Figures 6C,D; Supplementary Figures 4A–F).

### Epigenetic Age Shift and Immunophenotype

**Figure 7:**
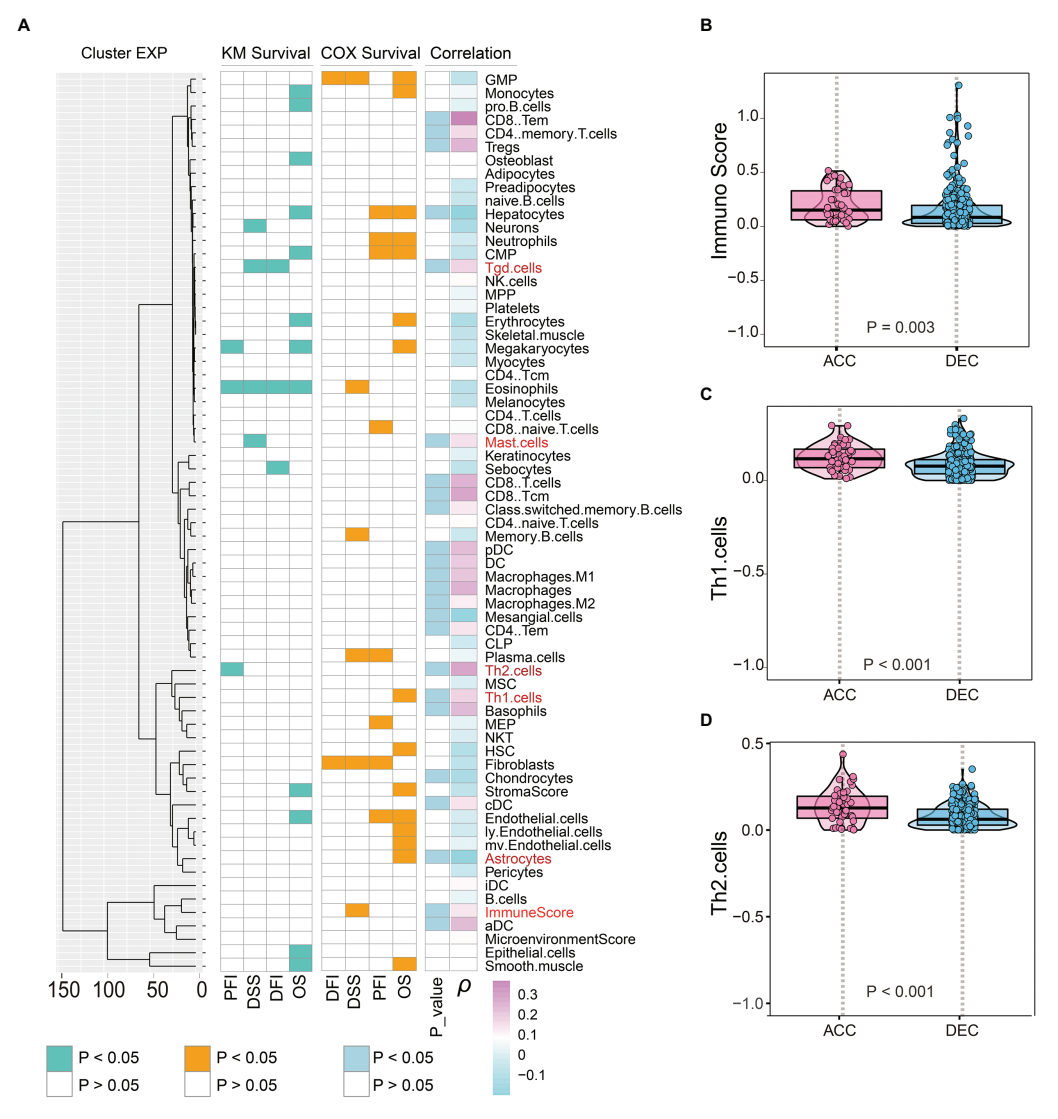
Epigenetic age and immune features in STAD patients. **(A)** Prognosis associated immune cells were shown on the left side of figure; Correlation between DNAm age and immune cells was shown on the right side of figure. DNAm age accelerated group showed higher immune score **(B)**, Th1 cells **(C)**, and Th2 cells **(D)** than DNAm age decelerated group. OS, overall survival; DSS, disease specific survival; PFI, progression free interval event; DFI, disease free interval event; S, results of Kaplan-Meier survival analysis; COX, results of univariate Cox regression analysis; ACC, DNAm age accelerated group; DEC, DNAm age decelerated group; *ρ*, correlation coefficient; STAD, stomach adenocarcinoma.

Enrichment analysis based on DEGs and WGCNA uncovered that epigenetic age shift was closely related to immune associated signaling and pathways in STAD patients. We then performed correlation analysis and found that DNAm age in TCGA STAD patients was significantly positively related to the infiltration intensity of CD8 T cell, CD8 effective memory T cell (CD8 Tem), CD8 central memory T cell (CD8 Tcm), Tgd cell, CD4 memory T cell, CD4 Tem, Th2 cell, Th1 cell, regulatory T cell, dendritic cell (DC), plasma DC (pDC), conventional DC (cDC), activated DC (aDC), macrophage, M1 macrophage, M2 macrophage, mast cell, and memory B cell (Figure 7A). We also found some DNAm age-associated immune cells including Tgd cell, Mast cell, Th2 cell, Th1 cell, and immune score are related to the prognosis of STAD patients (Figure 7A). In addition, the immune score, Th1 cell, Th2 cell, Tgd cell, and mast cell in DNAmAge-ACC group were significantly higher than DNAmAge-DEC group (Figures 7B–D; Supplementary Figure 5), which indicated that epigenetic age acceleration in STAD patients presented an immune-active pattern.

### Epigenetic Age Shift and Tumor Stemness Features

**Figure 8:**
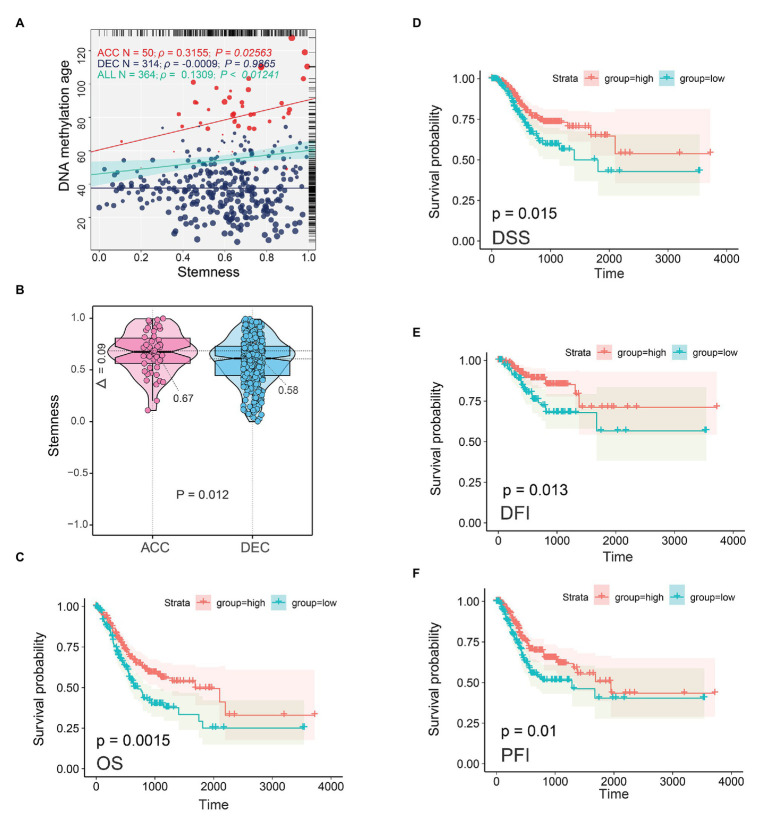
Epigenetic age and tumor stemness features in STAD patients. **(A)** Correlations between DNAm age and mRNAsi in normal stomach and STAD samples. **(B)** DNAm age-accelerated group showed higher mRNAsi than DNAm age decelerated group. Two-tailed statistical *p* values were calculated by two independent samples *t-test*. mRNAsi was related to the prognosis of STAD patients including OS **(C)**, DSS **(D)**, DFI **(E)**, and PFI **(F)**. ACC, DNAm age accelerated group; DEC, DNAm age decelerated group; OS, overall survival; DSS, disease specific survival; PFI, progression free interval event; DFI, disease free interval event; STAD, stomach adenocarcinoma.

DNA methylation age of embryonic and iPS cells was close to zero (Horvath, 2013), which indicated that epigenetic age shift may be related to the stemness feature of cancer. In addition, our GSEA results based on enriched motif-associated TFs showed that RUNX1 (TFs) in TCGA STAD cohort was significantly enriched in stem cells related pathway including *Wnt* and *Notch* signaling pathway (Figure 5B). We next sought to determine the relationship between DNAm age and stemness feature (mRNAsi) in TCGA STAD cohort. As showed in Figure 8A, in TCGA STAD patients, mRNAsi value was significantly positively correlated with DNAm age (*N* = 364, *r* = 0.1309, *p* = 0.0124). Interestingly, mRNAsi in DNAmAge-ACC group was significantly positively correlated with DNAm age (*N* = 50, *r* = 0.3155, *p* = 0.0256), but mRNAsi was not associated with DNAm age in DNAmAge-DEC patients (*N* = 314, *r* = −0.0009, *p* = 0.9865). In addition, compared with DNAmAge-DEC group, the mRNAsi in DNAmAge-ACC group was significantly increased (*p* = 0.012; independent samples t-test; Figure 8B). We also found that mRNAsi was related to the prognosis of STAD patients, including OS (*p* = 0.0015), DSS (*p* = 0.015), disease-free interval event (DFI, *p* = 0.013), and progression-free interval event (PFI, *p* = 0.01; Figures 8C–F).

## Discussion

An increasing number of studies suggest that DNAm age shift is a promising biomarker for studying aging associated diseases and cancer (Horvath, 2013; Zheng et al., 2016; Gao et al., 2018; Hofstatter et al., 2018; Rezwan et al., 2020; Sibbett et al., 2020). GC continues to be one of the leading causes of cancer-related death over the past 30 years (Dicken et al., 2005). We, therefore, focused on STAD and systematically analyzed the associations of DNAm age with clinical outcomes and other molecular characteristics in TCGA STAD patients.

Some previous studies suggested that a younger DNAm age in normal tissue is related to improved body health (Perna et al., 2016; Yang et al., 2016; Zhang et al., 2017). Interestingly, in this study, the epigenetic age pattern observed in normal gastric samples was disrupted in STAD samples and a younger DNAm age was related to worse prognosis of STAD patients. Additionally, compared with tumor adjacent normal tissues, the DNAm age in STAD tissues was significantly decreased which indicated that DNAm age shift in gastric cells inversely disrupted physiological epigenetic age pattern. Our analysis results were reasonable for DNAm age in normal tissue was correlated with the number of cell passages and caused a highly heritable measure of chronological age acceleration (Horvath, 2013). Notably, subclonal evolution, formation of cancer stem cells, and somatic cell mutations during the process of tumorigenesis might impair the proliferation, propagation, and epigenetic pattern of normal cells (Chen et al., 2015; van Niekerk et al., 2017). DNAm age of tumor cells also increases with cell propagation and tumor cells with lower DNAm age have a higher cell proliferation potential, thus might grow into more aggressive cancer cells (Horvath, 2013). Function annotation analysis found that DMPs and DEGs between DNAmAge-ACC and DNAmAge-DEC groups were significantly enriched in cancer associated pathway including immune, TP53, VEGFA, and hypoxia pathway, which suggested that decreased DNAm age may affect the prognosis of STAD patients by directly activating cancer-related signaling pathways. In addition, motif enrichment and survival analysis based on DMPs showed that Sox family associated motifs and transcription factors including Sox5, Sox21, Sox7, and Sox2 were related to the prognosis of STAD patients. It is relevant to know that Sox gene family are indeed crucial as deregulation of Sox-family transcription factors can potentially lead to changes in cell fate as well as irregular cell growth (Sarkar and Hochedlinger, 2013; Kumar and Mistri, 2019). Most STAD samples in DNAmAge-ACC group showed higher mean methylation level, which may affect the binding of Sox family transcription factors to regulatory elements and irregular cell growth. The present findings are in agreement with a previous study, which reported that epigenetic age pattern was impaired in CSCC and a younger DNAm age was related to worse prognosis of CSCC patients (Lu et al., 2020). Similarly, another study focused on breast cancer found that DNAm age acceleration was significantly associated with better prognosis after adjusting for clinicopathological variables (Ren et al., 2018). Combining these studies, we believe that DNAm age shift may share some common pathogenesis in different cancers.

The immune environment in tumors plays an important role in response to therapy and prognosis in various cancer types (Thorsson et al., 2019). Immune cell infiltration such as Th2 and mast cells has been reported to be associated with the prognosis of STAD patients (Wang et al., 2020). In this study, we also found that Tgd cell, mast cell, Th2 cell, Th1 cell, and summary immune score are related to the prognosis of STAD patients. In addition, DNAm age in TCGA STAD patients was significantly positively related to the infiltration intensity of immune cells and DNAmAge-ACC group presented an immune-active pattern. Such finding was reasonable for both DNAm age acceleration and immunoactivation in STAD patients and were associated with favorable prognosis. It is relevant to know that inflammatory processes exist in cancer microenvironment is considered to play a crucial role in regulating growth initiation and metastatic properties in cancer cells (Elinav et al., 2013). Recently study showed that STAD metastasis is enhanced not only by EMT process but also by the chronic inflammation exist in the cancer microenvironment (Ma et al., 2016). As we mentioned in the previous section, DMPs and DEGs between DNAmAge-ACC and DNAmAge-DEC groups were significantly enriched in immune-related pathway. We, therefore, speculate that DNAm age shift affects the prognosis of STAD patients by affecting the expression of immune-related genes.

Tumor microenvironment is composed of a highly integrated ecosystem including cancer cells, infiltrating immune cells, and cancer stem cells (CSCs; Malta et al., 2018). CSCs are cancer cells that share the characteristics of normal stem cells (NSCs) and thus have the ability to give rise to all cancer cell types (Bjerkvig et al., 2005). Unlike NSCs, CSCs are considered to be responsible for carcinogenesis and maintaining cancer cell growth, and play an important role in the process of cancer metastasis and recurrence (Kabakov et al., 2020; Schulte et al., 2020). Stemness was considered as the potential for cell differentiation from the cell-of-origin and self-renewal, and was originally attributed to NSCs that have the potential to differentiate into various types of cells in the adult organism (Barbee and Trippodo, 1987; Lian et al., 2019). In the present study, we calculated the mRNAsi (stemness) of STAD samples based on OCLR algorithm, and found that higher mRNAsi was related to a better prognosis of STAD patients including OS, DSS, DFI, and PFI. In addition, compared to the DNAmAge-DEC group, mRNAsi in DNAmAge-ACC group was significantly increased and was significantly positively correlated with DNAm age. Such findings were reasonable for both DNAmAge-ACC and higher mRNAsi in STAD patients were associated with favorable prognosis. Interestingly, early study showed that the relationship between mRNAsi and OS was different across cancer-derived (Malta et al., 2018). On one hand, higher mRNAsi in adrenocortical carcinoma (ACC), kidney chromophobe (KICH), liver hepatocellular carcinoma (LIHC), and prostate adenocarcinoma (PRAD) plays a harmful role in the patient’s OS; however, higher mRNAsi in colon adenocarcinoma (COAD), glioblastoma multiforme lower grade glioma (GB-LGG), and STAD plays a protective role in the patient’s OS (Malta et al., 2018). This phenomenon indicates that mRNAsi may be a double edged sword with various biological effects in different tumors. The association between tumor stemness signatures and adverse prognosis outcome for some cancer types, including STAD, may reflect cancer cell origins or the impact of their microenvironment. Dedifferentiated cells can arise from different sources: from progenitor cells or long lived stem cells that accumulate mutations in oncogenic pathways, or *via* dedifferentiation from non-stem cancer cells that convert to cancer stem cells through deregulation of developmental and/or non-developmental pathways. We speculate that the inherent stemness of cancer stem cells and dedifferentiation induced by the tumor microenvironment may be the cause of the difference in the prognosis of each tumor.

We would like to acknowledge that our present study is not devoid of limitations. First, our present study has a drawback of retrospective design with selection bias, and it is also limited to a single center (TCGA) that provided all of the STAD patient samples. Second, we simply mixed truly gastric tissue samples with two adjacent-normal gastric samples collected from TCGA STAD cohort. Due to the lack of sufficient TCGA cohort adjacent-normal gastric samples, we cannot definitively confirm whether there is a difference between adjacent-normal and normal gastric tissue. Finally, our findings will need further confirmation from other research centers and larger prospective studies for their generalizability.

## Conclusion

In summary, this study revealed novel evidence that epigenetic clock shift was associated with canceration and all-cause mortality. DNAm age acceleration in STAD patients was associated with higher mRNAsi, immunoactivation, and favorable prognosis. DNAm age shift could be considered as a useful indicator to guide clinical prognosis assessment and be a potential therapeutic target for STAD patients. Further molecular biology experiments *in vivo* and *in vitro* are needed to investigate the mechanism of age-associated DNAm patterns in STAD. In addition, an external cohort to validate the prognosis effect of DNAm age shift is warranted.

## Data Availability Statement

The datasets presented in this study can be found in online repositories. The names of the repository/repositories and accession number(s) can be found in the article/Supplementary Material.

## Author Contributions

CZ, CH, and SY designed the study and research. QW, SZ, WW, JC, and DZ collected the data. ML, LL, JL, HY, and HC completed the data analysis and interpretation. CZ, CH, and QZ drafted and revised the article for important intellectual content. All authors contributed to the article and approved the submitted version.

### Conflict of Interest

The authors declare that the research was conducted in the absence of any commercial or financial relationships that could be construed as a potential conflict of interest.

## Footnotes

^1^https://xenabrowser.net/datapages/

^2^https://www.cbioportal.org/

^3^https://www.ncbi.nlm.nih.gov/gds/

^4^http://software.broadinstitute.org/gsea/msigdb/index.jsp
