# Supplementary material for: Epigenetic Age Acceleration of Stomach Adenocarcinoma Associated With Tumor Stemness Features, Immunoactivation, and Favorable Prognosis
Source: Front Genet. 2021 Mar 18;12:563051. doi: 10.3389/fgene.2021.563051 (PMC8012546; doi:10.3389/fgene.2021.563051)
Supplement: Supplementary file 1 [file Data_Sheet_1.docx]

Supplementary Material

## Supplementary Figures





**Supplementary Figure 1.** Prognostic value of DNAm age and epigenetic age shifts in STAD patients. In normal stomach tissues, there was no difference between DNAm age and chronological age. Two-tailed statistical P values were calculated by a paired samples t-test (A). Patients with higher DNAm age showed significantly favourable prognosis of OS by Kaplan-Meier estimator with log-rank test (B). However, DNAm age showed no significant correlation with DSS (C), DFI (D), and PFI (E) in STAD patients. Patients with higher DNAm age shift value showed significantly better prognosis of DSS (G), but not OS (F), DFI (H), and PFI (I). DNAm age shift value was further discretized into quartiles: Q1, Q2, Q3, Q4, and rate of DSS was also well distinguished (K). OS represent overall survival; DSS represent disease specific survival; PFI represent progression free interval event; DFI represent disease free interval event; STAD represent stomach adenocarcinoma.


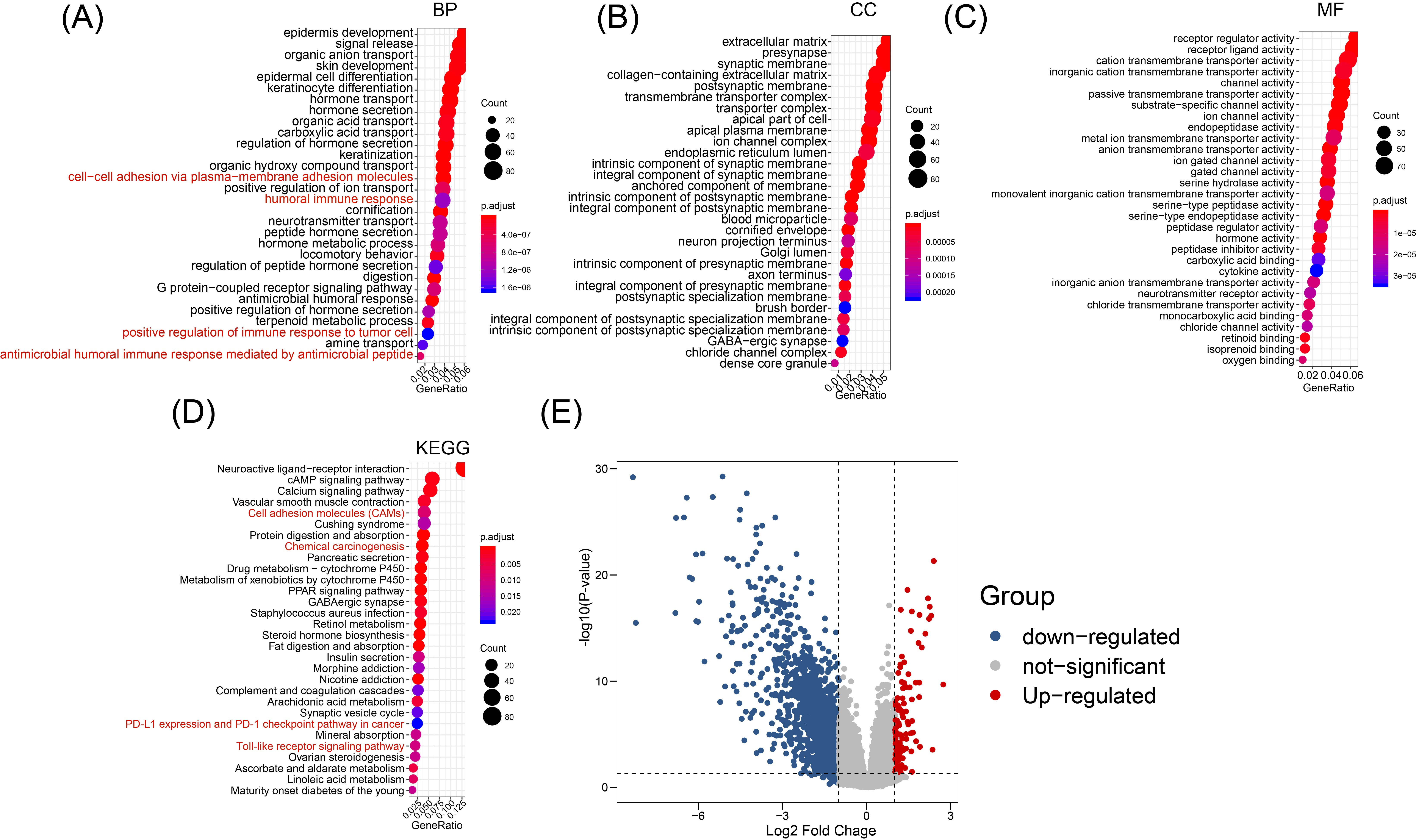


**Supplementary Figure 2.** Functional and pathway enrichment analysis of differential expression genes (DEGs) between DNAm age accelerated and DNAm age decelerated group. Biological process (BP) (A), cell component (CC) (B), molecular function (MF) (C), and Kyoto encyclopedia of genes and genomes (KEGG) (D) pathway of DEGs in TCGA STAD cohort. Using edgeR package, we identified 1782 DEGs in samples of 70 DNAm age accelerated (DNAmAge-ACC) and 318 DNAm age decelerated (DNAmAge-DEC) samples. STAD represent stomach adenocarcinoma; TCGA represent the cancer genome atlas; P < 0.05 was set as the cutoff criteria.


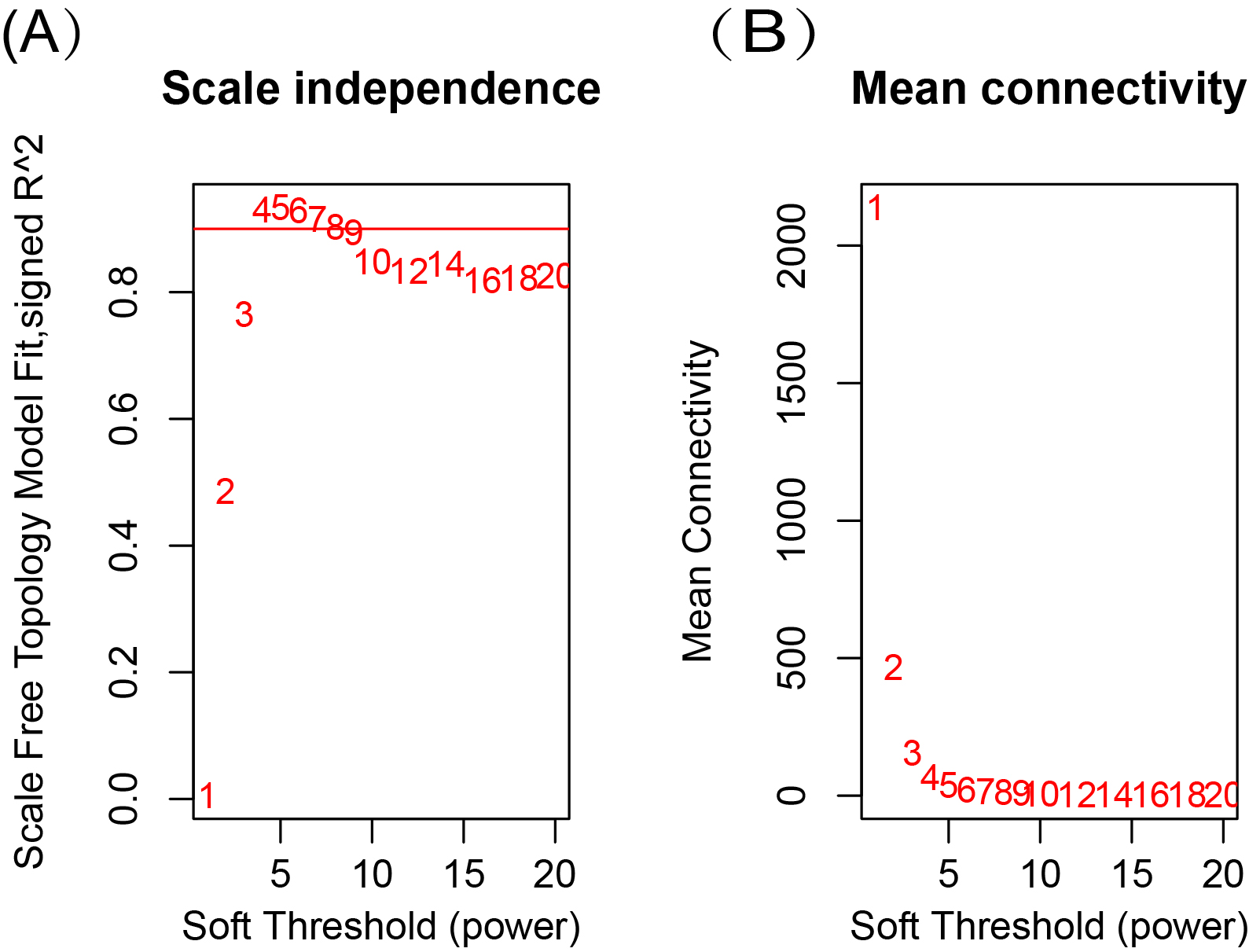


**Supplementary Figure 3.** Weighted gene co-expression network analysis. (A) Displays the influence of soft‐thresholding power (x‐axis) on the scale‐free fit index (y‐axis). The red line parallel to the x‐axis represents scale‐free fit index of 0.9. (B) Analysis of the mean connectivity for various soft‐thresholding powers; X‐axis represents soft‐thresholding power; Y‐axis represents mean connectivity (or degree).





**Supplementary Figure 4.** Functional and pathway enrichment analysis of genes in top 2 DNAm-age associated gene modules. Biological process (BP) (A), cell component (CC) (B), molecular function (MF) (C) of genes in saddlebrown module. BP (D), MF (E), and CC (F) of genes in lightgreen module. *P < 0.05* was set as the cutoff criteria.


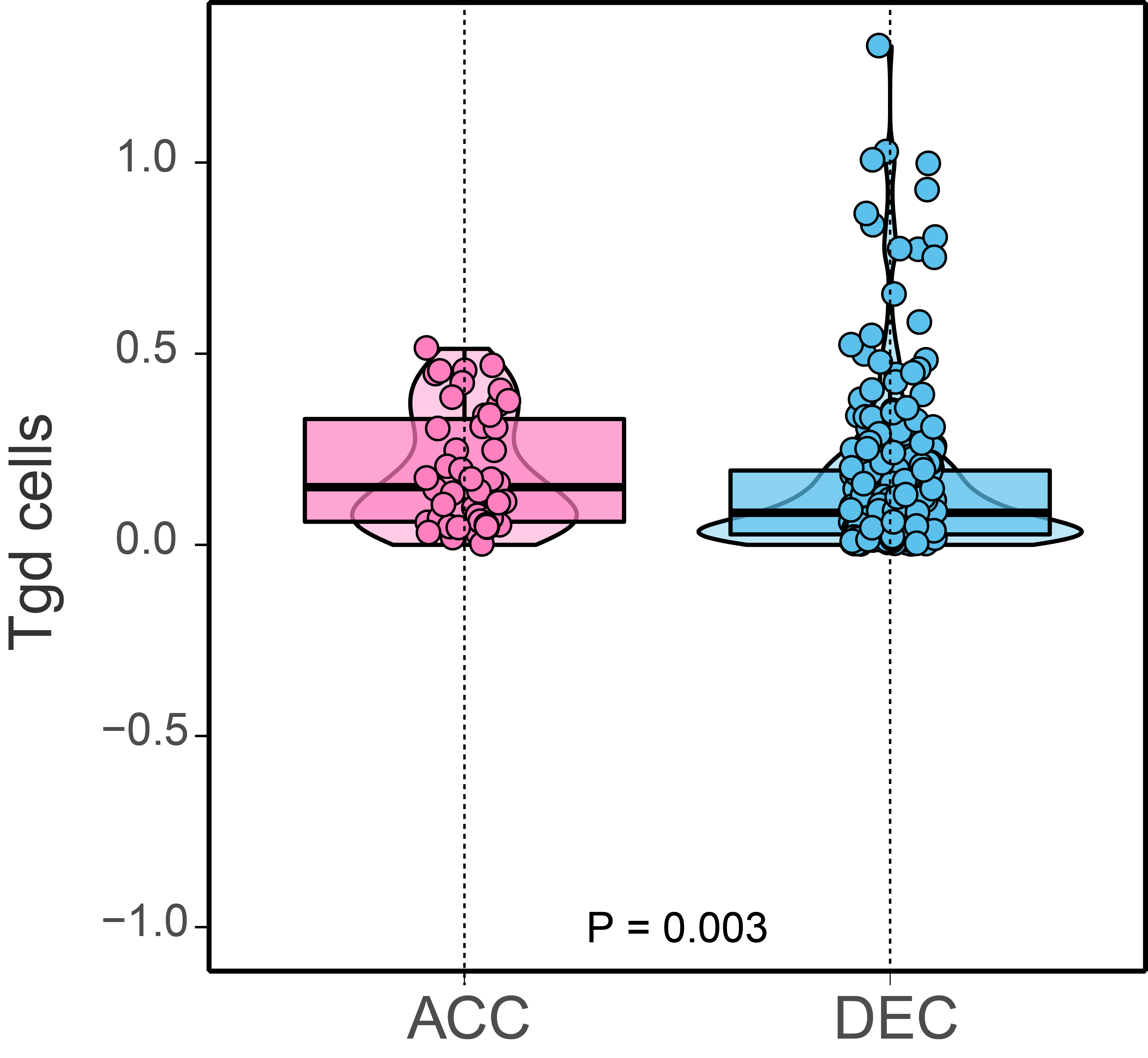


**Supplementary Figure 5.** DNAm age accelerated group showed higher Tgd cells than DNAm age decelerated group. Two-tailed statistical *P values* were calculated by a paired samples t-test.

## Supplementary tables

**Supplementary table1.** Significantly enriched motifs in different methylation sites

| **Motif** | **Percentage** | **lowerOR** | **upperOR** | **OR** | **p.value** |
| --- | --- | --- | --- | --- | --- |
| MAFG_HUMAN.H11MO.1.A | 0.09762698 | 1.208548 | 1.283094 | 1.245174 | 2.05E-48 |
| MAFK_HUMAN.H11MO.1.A | 0.11193847 | 1.165491 | 1.23214 | 1.198338 | 2.89E-38 |
| MAF_HUMAN.H11MO.0.A | 0.07195499 | 1.207949 | 1.29437 | 1.250299 | 3.14E-38 |
| ANDR_HUMAN.H11MO.1.A | 0.08829496 | 1.185958 | 1.262225 | 1.223408 | 4.76E-38 |
| RUNX2_HUMAN.H11MO.0.A | 0.10139343 | 1.165332 | 1.235146 | 1.199652 | 1.35E-35 |
| BACH2_HUMAN.H11MO.0.A | 0.08275486 | 1.178031 | 1.256068 | 1.216309 | 4.32E-34 |
| ZN260_HUMAN.H11MO.0.C | 0.03363812 | 1.29352 | 1.433533 | 1.361441 | 6.70E-34 |
| PITX2_HUMAN.H11MO.0.D | 0.05838252 | 1.213826 | 1.310321 | 1.261029 | 6.95E-34 |
| FIGLA_HUMAN.H11MO.0.D | 0.10766745 | 1.154534 | 1.221577 | 1.187556 | 9.71E-34 |
| MCR_HUMAN.H11MO.0.D | 0.05900941 | 1.201514 | 1.296213 | 1.247813 | 1.77E-31 |
| ZNF8_HUMAN.H11MO.0.C | 0.03324057 | 1.278879 | 1.417547 | 1.346158 | 2.20E-31 |
| RXRB_HUMAN.H11MO.0.C | 0.11714728 | 1.140828 | 1.204338 | 1.172153 | 2.38E-31 |
| NF2L2_HUMAN.H11MO.0.A | 0.05998288 | 1.198391 | 1.291918 | 1.244153 | 3.43E-31 |
| IRX3_HUMAN.H11MO.0.D | 0.02964741 | 1.296782 | 1.447086 | 1.369551 | 3.84E-31 |
| PEBB_HUMAN.H11MO.0.C | 0.10070538 | 1.149447 | 1.218176 | 1.183228 | 1.15E-30 |
| RUNX1_HUMAN.H11MO.0.A | 0.07972743 | 1.166972 | 1.245347 | 1.205432 | 2.52E-30 |
| P53_HUMAN.H11MO.0.A | 0.07520667 | 1.169592 | 1.250588 | 1.209299 | 1.09E-29 |
| TGIF2_HUMAN.H11MO.0.D | 0.07482952 | 1.168667 | 1.249757 | 1.208421 | 2.29E-29 |
| ZFP42_HUMAN.H11MO.0.A | 0.07616485 | 1.166693 | 1.246954 | 1.206078 | 2.87E-29 |
| GCR_HUMAN.H11MO.1.A | 0.08170494 | 1.160455 | 1.237373 | 1.1982 | 3.45E-29 |
| ATF2_HUMAN.H11MO.0.B | 0.0842329 | 1.156766 | 1.232193 | 1.193804 | 7.22E-29 |
| GCR_HUMAN.H11MO.0.A | 0.07703638 | 1.160638 | 1.239812 | 1.199471 | 5.25E-28 |
| ANDR_HUMAN.H11MO.2.A | 0.07780598 | 1.159433 | 1.238101 | 1.198022 | 6.48E-28 |
| MAFF_HUMAN.H11MO.0.B | 0.06307147 | 1.176236 | 1.265218 | 1.219788 | 1.52E-27 |
| NFE2_HUMAN.H11MO.0.A | 0.09865142 | 1.139286 | 1.207891 | 1.173023 | 2.20E-27 |
| Z354A_HUMAN.H11MO.0.C | 0.06199097 | 1.176846 | 1.266695 | 1.220819 | 2.50E-27 |
| HXC10_HUMAN.H11MO.0.D | 0.0605588 | 1.177909 | 1.268904 | 1.22245 | 4.38E-27 |
| MAFB_HUMAN.H11MO.0.B | 0.07767856 | 1.15569 | 1.234069 | 1.194152 | 5.17E-27 |
| PRGR_HUMAN.H11MO.0.A | 0.06038551 | 1.177702 | 1.268806 | 1.222294 | 5.56E-27 |
| RUNX3_HUMAN.H11MO.0.A | 0.07326993 | 1.159969 | 1.241084 | 1.199718 | 6.75E-27 |
| OZF_HUMAN.H11MO.0.C | 0.03464216 | 1.240458 | 1.370487 | 1.303599 | 1.02E-26 |
| RARG_HUMAN.H11MO.1.B | 0.12855876 | 1.120235 | 1.179636 | 1.14956 | 1.24E-26 |
| RXRA_HUMAN.H11MO.1.A | 0.11572021 | 1.125983 | 1.188776 | 1.156955 | 1.61E-26 |
| FOXD2_HUMAN.H11MO.0.D | 0.0441118 | 1.208342 | 1.318936 | 1.262246 | 1.67E-26 |
| ZN214_HUMAN.H11MO.0.C | 0.03589595 | 1.233155 | 1.3598 | 1.294721 | 2.25E-26 |
| P73_HUMAN.H11MO.0.A | 0.08939074 | 1.14156 | 1.21367 | 1.176974 | 4.01E-26 |
| PIT1_HUMAN.H11MO.0.C | 0.03283794 | 1.243617 | 1.377861 | 1.30877 | 4.16E-26 |
| BHE22_HUMAN.H11MO.0.D | 0.02589625 | 1.278306 | 1.436642 | 1.354811 | 7.23E-26 |
| ZNF85_HUMAN.H11MO.0.C | 0.04075818 | 1.212935 | 1.328772 | 1.269357 | 9.64E-26 |
| ATF2_HUMAN.H11MO.1.B | 0.04192023 | 1.20837 | 1.321989 | 1.263763 | 1.44E-25 |
| NR1H3_HUMAN.H11MO.0.B | 0.09646494 | 1.134084 | 1.203002 | 1.167958 | 1.48E-25 |
| BHE23_HUMAN.H11MO.0.D | 0.05928463 | 1.171693 | 1.262986 | 1.216366 | 1.98E-25 |
| ANDR_HUMAN.H11MO.0.A | 0.07765308 | 1.148208 | 1.22596 | 1.18637 | 3.13E-25 |
| OLIG1_HUMAN.H11MO.0.D | 0.0269054 | 1.264326 | 1.417018 | 1.338179 | 5.69E-25 |
| MAF_HUMAN.H11MO.1.B | 0.06643018 | 1.158069 | 1.242968 | 1.19966 | 1.21E-24 |
| FOXH1_HUMAN.H11MO.0.A | 0.05221553 | 1.179138 | 1.277315 | 1.227104 | 1.48E-24 |
| MITF_HUMAN.H11MO.0.A | 0.1377124 | 1.10893 | 1.165873 | 1.137018 | 3.00E-24 |
| ZN250_HUMAN.H11MO.0.C | 0.04064096 | 1.203045 | 1.317773 | 1.258919 | 4.03E-24 |
| DDIT3_HUMAN.H11MO.0.D | 0.04002426 | 1.203399 | 1.319044 | 1.259729 | 6.48E-24 |
| JUNB_HUMAN.H11MO.0.A | 0.0685453 | 1.150908 | 1.233813 | 1.191545 | 1.10E-23 |
| PRGR_HUMAN.H11MO.1.A | 0.12291673 | 1.112494 | 1.17263 | 1.142164 | 1.46E-23 |
| TF2LX_HUMAN.H11MO.0.D | 0.05592592 | 1.167431 | 1.26097 | 1.213173 | 1.49E-23 |
| FOXB1_HUMAN.H11MO.0.D | 0.04295995 | 1.192995 | 1.303195 | 1.246719 | 1.64E-23 |
| MEIS2_HUMAN.H11MO.0.B | 0.05629288 | 1.166382 | 1.259505 | 1.211943 | 1.71E-23 |
| TYY1_HUMAN.H11MO.0.A | 0.09186773 | 1.129137 | 1.199288 | 1.163626 | 1.81E-23 |
| FOXG1_HUMAN.H11MO.0.D | 0.10821789 | 1.118828 | 1.183062 | 1.150486 | 2.20E-23 |
| ZFP28_HUMAN.H11MO.0.C | 0.10839118 | 1.117786 | 1.181816 | 1.149346 | 4.15E-23 |
| MAFG_HUMAN.H11MO.0.A | 0.05737337 | 1.162322 | 1.254106 | 1.207235 | 4.79E-23 |
| TEF_HUMAN.H11MO.0.D | 0.03770527 | 1.20431 | 1.323772 | 1.262428 | 5.16E-23 |
| ZN274_HUMAN.H11MO.0.A | 0.06155265 | 1.155993 | 1.243965 | 1.199067 | 5.99E-23 |
| RORG_HUMAN.H11MO.0.C | 0.05891767 | 1.158646 | 1.248867 | 1.202782 | 8.93E-23 |
| HAND1_HUMAN.H11MO.0.D | 0.12067929 | 1.110332 | 1.170836 | 1.140178 | 1.15E-22 |
| THA_HUMAN.H11MO.1.D | 0.10886517 | 1.115834 | 1.179663 | 1.14726 | 1.16E-22 |
| ARI3A_HUMAN.H11MO.0.D | 0.05800027 | 1.159075 | 1.250067 | 1.203575 | 1.41E-22 |
| JUND_HUMAN.H11MO.0.A | 0.07032914 | 1.143487 | 1.224644 | 1.183305 | 1.50E-22 |
| FUBP1_HUMAN.H11MO.0.D | 0.08071109 | 1.132712 | 1.207645 | 1.169513 | 2.64E-22 |
| MAFF_HUMAN.H11MO.1.B | 0.09986443 | 1.119228 | 1.185963 | 1.152088 | 2.80E-22 |
| BATF_HUMAN.H11MO.1.A | 0.03621194 | 1.203142 | 1.324936 | 1.262385 | 3.37E-22 |
| TWST1_HUMAN.H11MO.1.A | 0.09272397 | 1.123177 | 1.192487 | 1.157239 | 3.83E-22 |
| TBX2_HUMAN.H11MO.0.D | 0.10639328 | 1.114961 | 1.179355 | 1.146655 | 4.53E-22 |
| JUN_HUMAN.H11MO.0.A | 0.06268412 | 1.150057 | 1.236663 | 1.192493 | 4.55E-22 |
| PO3F3_HUMAN.H11MO.0.D | 0.05099232 | 1.166092 | 1.263968 | 1.213899 | 7.75E-22 |
| NR1D1_HUMAN.H11MO.1.D | 0.0318237 | 1.215252 | 1.347444 | 1.279405 | 9.22E-22 |
| TFE3_HUMAN.H11MO.0.B | 0.11254498 | 1.109781 | 1.172165 | 1.140523 | 1.61E-21 |
| FOXL1_HUMAN.H11MO.0.D | 0.08942642 | 1.122345 | 1.192794 | 1.156954 | 2.05E-21 |
| OLIG3_HUMAN.H11MO.0.D | 0.02484124 | 1.244943 | 1.400842 | 1.32024 | 2.28E-21 |
| ARI5B_HUMAN.H11MO.0.C | 0.03445868 | 1.201421 | 1.326205 | 1.262059 | 3.50E-21 |
| FOXA1_HUMAN.H11MO.0.A | 0.06006442 | 1.148504 | 1.236761 | 1.191731 | 3.94E-21 |
| THA_HUMAN.H11MO.0.C | 0.13040376 | 1.101035 | 1.15883 | 1.129529 | 4.26E-21 |
| ONEC2_HUMAN.H11MO.0.D | 0.04554397 | 1.171753 | 1.276092 | 1.222676 | 4.73E-21 |
| ZN382_HUMAN.H11MO.0.C | 0.06280644 | 1.144492 | 1.230483 | 1.186596 | 4.86E-21 |
| THB_HUMAN.H11MO.1.D | 0.12925701 | 1.101214 | 1.15919 | 1.129824 | 4.95E-21 |
| DMRT1_HUMAN.H11MO.0.D | 0.03569208 | 1.195154 | 1.316785 | 1.254295 | 6.98E-21 |
| LMX1A_HUMAN.H11MO.0.D | 0.04431567 | 1.172856 | 1.278826 | 1.224514 | 7.87E-21 |
| HXD13_HUMAN.H11MO.0.D | 0.04379581 | 1.173714 | 1.280473 | 1.225787 | 8.60E-21 |
| FOS_HUMAN.H11MO.0.A | 0.06449344 | 1.1411 | 1.225601 | 1.182501 | 9.14E-21 |
| FOXP1_HUMAN.H11MO.0.A | 0.07322916 | 1.131259 | 1.209659 | 1.169733 | 1.45E-20 |
| FOSB_HUMAN.H11MO.0.A | 0.06275038 | 1.141975 | 1.227726 | 1.183974 | 1.55E-20 |
| FOSL2_HUMAN.H11MO.0.A | 0.06275547 | 1.139503 | 1.224979 | 1.181411 | 4.67E-20 |
| NDF1_HUMAN.H11MO.0.A | 0.14005178 | 1.094116 | 1.149626 | 1.121503 | 5.17E-20 |
| BPTF_HUMAN.H11MO.0.D | 0.09139884 | 1.114696 | 1.183838 | 1.148685 | 6.18E-20 |
| MEIS1_HUMAN.H11MO.1.B | 0.12370162 | 1.099081 | 1.158109 | 1.128188 | 6.85E-20 |
| ZKSC1_HUMAN.H11MO.0.B | 0.11630633 | 1.101619 | 1.162513 | 1.131615 | 8.74E-20 |
| FOXA2_HUMAN.H11MO.0.A | 0.05746002 | 1.144099 | 1.233911 | 1.188039 | 1.07E-19 |
| TAL1_HUMAN.H11MO.1.A | 0.16709989 | 1.085883 | 1.136859 | 1.111082 | 1.23E-19 |
| MAFK_HUMAN.H11MO.0.A | 0.04701181 | 1.159542 | 1.260724 | 1.208952 | 1.47E-19 |
| CPEB1_HUMAN.H11MO.0.D | 0.10695901 | 1.104286 | 1.167703 | 1.135514 | 1.98E-19 |
| OLIG2_HUMAN.H11MO.0.B | 0.14439416 | 1.090368 | 1.14489 | 1.117292 | 2.68E-19 |
| RORA_HUMAN.H11MO.0.C | 0.11930318 | 1.098102 | 1.158033 | 1.127646 | 3.62E-19 |
| BRAC_HUMAN.H11MO.1.B | 0.07403443 | 1.123595 | 1.200874 | 1.161519 | 3.94E-19 |
| FOSL1_HUMAN.H11MO.0.A | 0.06516111 | 1.131473 | 1.214536 | 1.17219 | 4.82E-19 |
| ERR1_HUMAN.H11MO.0.A | 0.08984944 | 1.111691 | 1.181116 | 1.145807 | 5.16E-19 |
| ZN335_HUMAN.H11MO.1.A | 0.07760721 | 1.119883 | 1.195107 | 1.156796 | 5.78E-19 |
| SOX2_HUMAN.H11MO.1.A | 0.04681304 | 1.155516 | 1.256443 | 1.204785 | 6.86E-19 |
| FOXA3_HUMAN.H11MO.0.B | 0.04750619 | 1.15367 | 1.253652 | 1.202498 | 8.45E-19 |
| FOXK1_HUMAN.H11MO.0.A | 0.07079804 | 1.124242 | 1.203333 | 1.163021 | 1.10E-18 |
| ZN232_HUMAN.H11MO.0.D | 0.02759854 | 1.205966 | 1.347013 | 1.274282 | 1.30E-18 |
| NR1H4_HUMAN.H11MO.1.B | 0.07812707 | 1.117627 | 1.192413 | 1.154337 | 1.43E-18 |
| RARB_HUMAN.H11MO.0.D | 0.14676412 | 1.087112 | 1.141057 | 1.113758 | 1.48E-18 |
| FOXC2_HUMAN.H11MO.0.D | 0.04575803 | 1.155204 | 1.257289 | 1.205029 | 1.52E-18 |
| BARX1_HUMAN.H11MO.0.D | 0.03543725 | 1.178312 | 1.298038 | 1.23654 | 1.62E-18 |
| ZN317_HUMAN.H11MO.0.C | 0.04244009 | 1.161356 | 1.268252 | 1.213473 | 1.72E-18 |
| FOXJ3_HUMAN.H11MO.0.A | 0.07797927 | 1.117066 | 1.191878 | 1.153794 | 1.92E-18 |
| P73_HUMAN.H11MO.1.A | 0.10603142 | 1.100642 | 1.164107 | 1.131907 | 1.95E-18 |
| OLIG2_HUMAN.H11MO.1.B | 0.12440496 | 1.092277 | 1.150693 | 1.121093 | 3.75E-18 |
| NKX61_HUMAN.H11MO.0.B | 0.02963212 | 1.193873 | 1.327768 | 1.258817 | 3.76E-18 |
| HXB2_HUMAN.H11MO.0.D | 0.04958564 | 1.146017 | 1.243016 | 1.19343 | 4.03E-18 |
| TBX19_HUMAN.H11MO.0.D | 0.03616097 | 1.172716 | 1.290403 | 1.22997 | 4.46E-18 |
| MEF2B_HUMAN.H11MO.0.A | 0.06438641 | 1.126792 | 1.209932 | 1.167564 | 4.93E-18 |
| EVI1_HUMAN.H11MO.0.B | 0.03491738 | 1.174313 | 1.29449 | 1.232738 | 7.70E-18 |
| LMX1B_HUMAN.H11MO.0.D | 0.03936169 | 1.162362 | 1.273652 | 1.216557 | 1.08E-17 |
| NRL_HUMAN.H11MO.0.D | 0.09696442 | 1.101581 | 1.167762 | 1.134148 | 1.24E-17 |
| NR1H3_HUMAN.H11MO.1.B | 0.09150587 | 1.103915 | 1.172157 | 1.137453 | 1.65E-17 |
| NANOG_HUMAN.H11MO.1.B | 0.04430548 | 1.150834 | 1.254112 | 1.201237 | 1.71E-17 |
| SOX7_HUMAN.H11MO.0.D | 0.02701752 | 1.197094 | 1.338237 | 1.265446 | 2.13E-17 |
| BATF_HUMAN.H11MO.0.A | 0.03225691 | 1.178379 | 1.30419 | 1.239504 | 2.26E-17 |
| NR4A3_HUMAN.H11MO.0.D | 0.07761231 | 1.111541 | 1.186056 | 1.148097 | 3.00E-17 |
| FOXF1_HUMAN.H11MO.0.D | 0.06687359 | 1.119508 | 1.200361 | 1.159146 | 4.07E-17 |
| NANOG_HUMAN.H11MO.0.A | 0.03894376 | 1.158578 | 1.269955 | 1.212826 | 4.30E-17 |
| STF1_HUMAN.H11MO.0.B | 0.14052068 | 1.083363 | 1.138073 | 1.110378 | 4.76E-17 |
| ZN490_HUMAN.H11MO.0.C | 0.05209321 | 1.135344 | 1.228732 | 1.180991 | 5.31E-17 |
| SOX1_HUMAN.H11MO.0.D | 0.02552419 | 1.199404 | 1.345312 | 1.26998 | 5.75E-17 |
| BRAC_HUMAN.H11MO.0.A | 0.06134369 | 1.123923 | 1.208751 | 1.165477 | 5.94E-17 |
| ZNF41_HUMAN.H11MO.0.C | 0.10198975 | 1.096001 | 1.160239 | 1.127603 | 6.94E-17 |
| TBX5_HUMAN.H11MO.0.D | 0.09040498 | 1.101015 | 1.169432 | 1.134646 | 9.33E-17 |
| TEAD1_HUMAN.H11MO.0.A | 0.06645566 | 1.117301 | 1.19822 | 1.156969 | 1.17E-16 |
| PO3F1_HUMAN.H11MO.0.C | 0.03053423 | 1.177212 | 1.306488 | 1.239951 | 1.37E-16 |
| ZN136_HUMAN.H11MO.0.C | 0.03545763 | 1.162965 | 1.28054 | 1.220165 | 1.45E-16 |
| CEBPG_HUMAN.H11MO.0.B | 0.03778682 | 1.157101 | 1.270007 | 1.212061 | 1.53E-16 |
| TEAD3_HUMAN.H11MO.0.D | 0.03992233 | 1.151462 | 1.260496 | 1.204601 | 2.21E-16 |
| NR2E3_HUMAN.H11MO.0.C | 0.0410385 | 1.148843 | 1.255997 | 1.201081 | 2.45E-16 |
| MEF2C_HUMAN.H11MO.0.A | 0.06561981 | 1.115706 | 1.196982 | 1.155546 | 3.10E-16 |
| SOX11_HUMAN.H11MO.0.D | 0.02709907 | 1.185453 | 1.324481 | 1.252761 | 3.45E-16 |
| NR6A1_HUMAN.H11MO.0.B | 0.05006473 | 1.132717 | 1.227642 | 1.179126 | 3.60E-16 |
| P63_HUMAN.H11MO.0.A | 0.11844184 | 1.086377 | 1.145698 | 1.115626 | 3.83E-16 |
| PO3F4_HUMAN.H11MO.0.D | 0.03419875 | 1.161642 | 1.281247 | 1.219786 | 5.14E-16 |
| ZIM3_HUMAN.H11MO.0.C | 0.07134848 | 1.109556 | 1.186915 | 1.14751 | 5.75E-16 |
| TBR1_HUMAN.H11MO.0.D | 0.05556915 | 1.124189 | 1.213342 | 1.16782 | 6.49E-16 |
| ATF4_HUMAN.H11MO.0.A | 0.03496325 | 1.158731 | 1.276583 | 1.216053 | 6.59E-16 |
| FOXD3_HUMAN.H11MO.0.D | 0.07031895 | 1.109891 | 1.187885 | 1.14818 | 6.81E-16 |
| FOXF2_HUMAN.H11MO.0.D | 0.05450394 | 1.124939 | 1.215051 | 1.169028 | 7.64E-16 |
| FOXJ2_HUMAN.H11MO.0.C | 0.07160841 | 1.108456 | 1.185692 | 1.146341 | 7.95E-16 |
| GSX2_HUMAN.H11MO.0.D | 0.03103371 | 1.168376 | 1.295263 | 1.229952 | 8.69E-16 |
| MEF2D_HUMAN.H11MO.0.A | 0.06319379 | 1.114977 | 1.197706 | 1.155515 | 1.04E-15 |
| ZN708_HUMAN.H11MO.0.C | 0.129573 | 1.080767 | 1.137336 | 1.108676 | 1.33E-15 |
| SOX21_HUMAN.H11MO.0.D | 0.02624283 | 1.183204 | 1.324278 | 1.251485 | 1.40E-15 |
| VDR_HUMAN.H11MO.1.A | 0.10343211 | 1.089282 | 1.152525 | 1.120422 | 1.63E-15 |
| SOX5_HUMAN.H11MO.0.C | 0.02663527 | 1.180919 | 1.32052 | 1.24852 | 1.64E-15 |
| MEF2A_HUMAN.H11MO.0.A | 0.06638941 | 1.111012 | 1.191339 | 1.150372 | 1.64E-15 |
| DMBX1_HUMAN.H11MO.0.D | 0.02743545 | 1.17731 | 1.314138 | 1.243601 | 1.80E-15 |
| ZNF85_HUMAN.H11MO.1.C | 0.0396624 | 1.14451 | 1.252971 | 1.197348 | 2.17E-15 |
| PPARA_HUMAN.H11MO.1.B | 0.08526243 | 1.09728 | 1.167273 | 1.131673 | 2.26E-15 |
| FOXD1_HUMAN.H11MO.0.D | 0.03829139 | 1.146898 | 1.257717 | 1.200877 | 2.32E-15 |
| PPARG_HUMAN.H11MO.1.A | 0.07174093 | 1.105512 | 1.182348 | 1.143226 | 2.70E-15 |
| ZN418_HUMAN.H11MO.0.C | 0.08045116 | 1.099623 | 1.171868 | 1.135102 | 2.70E-15 |
| P5F1B_HUMAN.H11MO.0.D | 0.03542705 | 1.151307 | 1.267289 | 1.207745 | 3.95E-15 |
| STAT6_HUMAN.H11MO.0.B | 0.06743932 | 1.107634 | 1.187088 | 1.146582 | 4.34E-15 |
| FOXM1_HUMAN.H11MO.0.A | 0.06502859 | 1.108443 | 1.189407 | 1.148128 | 6.82E-15 |
| NR5A2_HUMAN.H11MO.0.B | 0.09487987 | 1.089552 | 1.155455 | 1.121969 | 8.53E-15 |
| TEAD4_HUMAN.H11MO.0.A | 0.05964649 | 1.112736 | 1.197633 | 1.154309 | 9.16E-15 |
| ZN333_HUMAN.H11MO.0.D | 0.02577903 | 1.176385 | 1.317674 | 1.244776 | 9.34E-15 |
| SRY_HUMAN.H11MO.0.B | 0.06320908 | 1.10915 | 1.191264 | 1.14937 | 9.76E-15 |
| NR2E1_HUMAN.H11MO.0.D | 0.03112035 | 1.158969 | 1.284178 | 1.219755 | 1.00E-14 |
| HSF4_HUMAN.H11MO.0.D | 0.06015616 | 1.110492 | 1.194842 | 1.151815 | 1.69E-14 |
| GATA5_HUMAN.H11MO.0.D | 0.02637024 | 1.17153 | 1.310304 | 1.238724 | 1.88E-14 |
| NKX32_HUMAN.H11MO.0.C | 0.03233846 | 1.152768 | 1.27468 | 1.212016 | 1.99E-14 |
| FOXC1_HUMAN.H11MO.0.C | 0.04425961 | 1.128639 | 1.229327 | 1.177787 | 2.24E-14 |
| P53_HUMAN.H11MO.1.A | 0.1138956 | 1.080216 | 1.14011 | 1.109708 | 2.46E-14 |
| ERR3_HUMAN.H11MO.0.B | 0.04850005 | 1.122258 | 1.21755 | 1.168814 | 2.64E-14 |
| HMBX1_HUMAN.H11MO.0.D | 0.03201227 | 1.152221 | 1.27466 | 1.211698 | 2.90E-14 |
| SPDEF_HUMAN.H11MO.0.D | 0.09140393 | 1.08822 | 1.155187 | 1.121147 | 3.53E-14 |
| ZSC16_HUMAN.H11MO.0.D | 0.04890778 | 1.120356 | 1.215012 | 1.166613 | 3.94E-14 |
| NKX31_HUMAN.H11MO.0.C | 0.03785817 | 1.137844 | 1.248097 | 1.191575 | 3.95E-14 |
| TWST1_HUMAN.H11MO.0.A | 0.04432586 | 1.126595 | 1.226897 | 1.175542 | 4.16E-14 |
| CDC5L_HUMAN.H11MO.0.D | 0.03260859 | 1.14857 | 1.269328 | 1.20727 | 5.01E-14 |
| IRF8_HUMAN.H11MO.0.B | 0.1125195 | 1.078639 | 1.138711 | 1.108256 | 6.69E-14 |
| COT2_HUMAN.H11MO.0.A | 0.10995586 | 1.079358 | 1.140195 | 1.109318 | 7.00E-14 |
| FOXJ3_HUMAN.H11MO.1.B | 0.08794328 | 1.088172 | 1.156385 | 1.12168 | 7.27E-14 |
| DLX1_HUMAN.H11MO.0.D | 0.03328135 | 1.144168 | 1.26301 | 1.201954 | 9.96E-14 |
| HNF1A_HUMAN.H11MO.0.C | 0.03097765 | 1.149401 | 1.273488 | 1.209676 | 1.14E-13 |
| FOXP3_HUMAN.H11MO.0.D | 0.03050875 | 1.15067 | 1.276034 | 1.211542 | 1.20E-13 |
| PO3F2_HUMAN.H11MO.0.A | 0.03617626 | 1.136215 | 1.248929 | 1.191053 | 1.55E-13 |
| DLX4_HUMAN.H11MO.0.D | 0.02934671 | 1.152786 | 1.281034 | 1.21499 | 1.56E-13 |
| HXD10_HUMAN.H11MO.0.D | 0.03591124 | 1.135633 | 1.248697 | 1.190637 | 2.15E-13 |
| SMCA1_HUMAN.H11MO.0.C | 0.06005933 | 1.103574 | 1.187293 | 1.144584 | 2.17E-13 |
| FOXO6_HUMAN.H11MO.0.D | 0.04614028 | 1.117857 | 1.215072 | 1.165337 | 2.83E-13 |
| PO2F1_HUMAN.H11MO.0.C | 0.03651265 | 1.133433 | 1.245173 | 1.187833 | 2.84E-13 |
| HLTF_HUMAN.H11MO.0.D | 0.08169985 | 1.088069 | 1.158688 | 1.122756 | 3.07E-13 |
| ZN549_HUMAN.H11MO.0.C | 0.06943213 | 1.095448 | 1.172538 | 1.133269 | 3.09E-13 |
| FOXQ1_HUMAN.H11MO.0.C | 0.0439589 | 1.120592 | 1.220565 | 1.169386 | 3.17E-13 |
| ZN418_HUMAN.H11MO.1.D | 0.05125735 | 1.111062 | 1.202476 | 1.155752 | 3.31E-13 |
| OVOL1_HUMAN.H11MO.0.C | 0.04959074 | 1.112846 | 1.206021 | 1.158355 | 3.61E-13 |
| PO2F3_HUMAN.H11MO.0.D | 0.02913265 | 1.14947 | 1.277666 | 1.211672 | 3.77E-13 |
| ALX4_HUMAN.H11MO.0.D | 0.02788396 | 1.152369 | 1.284052 | 1.216212 | 4.51E-13 |
| MNX1_HUMAN.H11MO.0.D | 0.04866314 | 1.112991 | 1.207095 | 1.158999 | 4.65E-13 |
| IRF4_HUMAN.H11MO.0.A | 0.11101088 | 1.075103 | 1.135373 | 1.104817 | 4.88E-13 |
| FOXO1_HUMAN.H11MO.0.A | 0.09020112 | 1.082752 | 1.149751 | 1.115693 | 5.13E-13 |
| HXA10_HUMAN.H11MO.0.C | 0.03440262 | 1.13531 | 1.250832 | 1.191501 | 5.17E-13 |
| PAX2_HUMAN.H11MO.0.D | 0.04056961 | 1.12322 | 1.227759 | 1.174176 | 6.83E-13 |
| ALX1_HUMAN.H11MO.0.B | 0.02610012 | 1.155656 | 1.292613 | 1.221972 | 7.60E-13 |
| CREM_HUMAN.H11MO.0.C | 0.10748397 | 1.075129 | 1.136282 | 1.105279 | 8.14E-13 |
| ZN680_HUMAN.H11MO.0.C | 0.07052282 | 1.091799 | 1.16802 | 1.129228 | 9.13E-13 |
| TBX4_HUMAN.H11MO.0.D | 0.05846406 | 1.100805 | 1.185357 | 1.142214 | 9.58E-13 |
| ERR2_HUMAN.H11MO.0.A | 0.06881033 | 1.092342 | 1.169525 | 1.130233 | 1.19E-12 |
| HXD11_HUMAN.H11MO.0.D | 0.04129334 | 1.119695 | 1.222869 | 1.169988 | 1.27E-12 |
| NKX23_HUMAN.H11MO.0.D | 0.04053393 | 1.12093 | 1.225194 | 1.171767 | 1.28E-12 |
| NR4A1_HUMAN.H11MO.0.A | 0.05956495 | 1.098522 | 1.182023 | 1.139426 | 1.52E-12 |
| PO5F1_HUMAN.H11MO.0.A | 0.03748611 | 1.124797 | 1.233892 | 1.177923 | 1.73E-12 |
| JDP2_HUMAN.H11MO.0.D | 0.07573163 | 1.085957 | 1.159024 | 1.12183 | 2.78E-12 |
| GRHL1_HUMAN.H11MO.0.D | 0.06988573 | 1.08926 | 1.165526 | 1.126686 | 2.83E-12 |
| ZN394_HUMAN.H11MO.0.C | 0.08815735 | 1.079674 | 1.14709 | 1.112831 | 2.85E-12 |
| SOX10_HUMAN.H11MO.0.B | 0.03785817 | 1.122037 | 1.230238 | 1.174721 | 3.08E-12 |
| HXD12_HUMAN.H11MO.0.D | 0.03721599 | 1.122635 | 1.231864 | 1.175815 | 3.58E-12 |
| SOX8_HUMAN.H11MO.0.D | 0.0288931 | 1.140235 | 1.267608 | 1.202035 | 3.65E-12 |
| FOXO4_HUMAN.H11MO.0.C | 0.04966209 | 1.105108 | 1.197345 | 1.150204 | 3.88E-12 |
| MEIS3_HUMAN.H11MO.0.D | 0.06557903 | 1.091326 | 1.170218 | 1.130019 | 3.99E-12 |
| HMX1_HUMAN.H11MO.0.D | 0.03355147 | 1.128925 | 1.245076 | 1.185417 | 4.13E-12 |
| HSF1_HUMAN.H11MO.1.A | 0.05913173 | 1.096024 | 1.179538 | 1.136939 | 4.18E-12 |
| HXA11_HUMAN.H11MO.0.D | 0.03177783 | 1.13253 | 1.252533 | 1.190836 | 4.38E-12 |
| ZNF41_HUMAN.H11MO.1.C | 0.0777805 | 1.083442 | 1.155376 | 1.118767 | 4.60E-12 |
| MYBB_HUMAN.H11MO.0.D | 0.0364362 | 1.122074 | 1.232425 | 1.175789 | 5.75E-12 |
| SRF_HUMAN.H11MO.0.A | 0.03130893 | 1.131861 | 1.252703 | 1.190562 | 6.75E-12 |
| DLX6_HUMAN.H11MO.0.D | 0.03062088 | 1.132817 | 1.255223 | 1.192257 | 8.02E-12 |
| CDX1_HUMAN.H11MO.0.C | 0.03177783 | 1.12982 | 1.249478 | 1.187952 | 8.84E-12 |
| GBX1_HUMAN.H11MO.0.D | 0.02639063 | 1.143298 | 1.277408 | 1.208261 | 8.93E-12 |
| HBP1_HUMAN.H11MO.0.D | 0.02035616 | 1.16489 | 1.323059 | 1.241118 | 1.02E-11 |
| ZN410_HUMAN.H11MO.0.D | 0.02757816 | 1.138671 | 1.268932 | 1.201789 | 1.21E-11 |
| SOX13_HUMAN.H11MO.0.D | 0.0315128 | 1.127516 | 1.247362 | 1.185736 | 1.73E-11 |
| DBP_HUMAN.H11MO.0.B | 0.03101842 | 1.128005 | 1.248919 | 1.186729 | 2.02E-11 |
| AIRE_HUMAN.H11MO.0.C | 0.02466285 | 1.143901 | 1.283017 | 1.21123 | 2.43E-11 |
| NR1I2_HUMAN.H11MO.0.C | 0.051344 | 1.097254 | 1.187069 | 1.141174 | 2.71E-11 |
| GATA6_HUMAN.H11MO.0.A | 0.03655852 | 1.114943 | 1.224141 | 1.16812 | 3.40E-11 |
| NR1I3_HUMAN.H11MO.0.C | 0.06092576 | 1.087946 | 1.169422 | 1.127874 | 4.00E-11 |
| ATF7_HUMAN.H11MO.0.D | 0.08683221 | 1.073833 | 1.141293 | 1.107019 | 4.03E-11 |
| PAX6_HUMAN.H11MO.0.C | 0.05104329 | 1.095506 | 1.185385 | 1.139452 | 4.98E-11 |
| HXC13_HUMAN.H11MO.0.D | 0.03266465 | 1.120546 | 1.237139 | 1.177215 | 5.07E-11 |
| ZN713_HUMAN.H11MO.0.D | 0.09431924 | 1.070234 | 1.134789 | 1.101993 | 5.49E-11 |
| NR4A2_HUMAN.H11MO.0.C | 0.06634863 | 1.08315 | 1.1608 | 1.121242 | 5.78E-11 |
| BATF3_HUMAN.H11MO.0.B | 0.04096205 | 1.105398 | 1.207159 | 1.155033 | 7.70E-11 |
| HXC12_HUMAN.H11MO.0.D | 0.03967769 | 1.106742 | 1.210356 | 1.157263 | 8.17E-11 |
| ALX3_HUMAN.H11MO.0.D | 0.02579942 | 1.134502 | 1.268815 | 1.199531 | 8.17E-11 |
| HXC6_HUMAN.H11MO.0.D | 0.03960633 | 1.106727 | 1.210436 | 1.157293 | 8.46E-11 |
| UNC4_HUMAN.H11MO.0.D | 0.02686462 | 1.131176 | 1.262083 | 1.194618 | 8.73E-11 |
| GSX1_HUMAN.H11MO.0.D | 0.02842421 | 1.12578 | 1.251999 | 1.187015 | 1.22E-10 |
| SOX9_HUMAN.H11MO.0.B | 0.04992202 | 1.093175 | 1.183777 | 1.137453 | 1.34E-10 |
| HLF_HUMAN.H11MO.0.C | 0.03523847 | 1.111505 | 1.22234 | 1.165441 | 1.38E-10 |
| GATA3_HUMAN.H11MO.0.A | 0.0576333 | 1.086297 | 1.169897 | 1.127247 | 1.46E-10 |
| ARX_HUMAN.H11MO.0.D | 0.02278218 | 1.140646 | 1.285255 | 1.210537 | 1.51E-10 |
| PROP1_HUMAN.H11MO.0.D | 0.02732332 | 1.126471 | 1.255508 | 1.189009 | 1.80E-10 |
| HSF2_HUMAN.H11MO.0.A | 0.05457529 | 1.087781 | 1.173862 | 1.129908 | 2.01E-10 |
| BRCA1_HUMAN.H11MO.0.D | 0.05280165 | 1.08903 | 1.176695 | 1.131911 | 2.20E-10 |
| HSF1_HUMAN.H11MO.0.A | 0.0762362 | 1.073579 | 1.145365 | 1.108825 | 2.60E-10 |
| NFAC3_HUMAN.H11MO.0.B | 0.04633396 | 1.094495 | 1.188844 | 1.140579 | 2.63E-10 |
| TCF7_HUMAN.H11MO.0.A | 0.0490352 | 1.091639 | 1.182907 | 1.136259 | 2.66E-10 |
| TLX1_HUMAN.H11MO.0.D | 0.08979848 | 1.067824 | 1.133736 | 1.100257 | 2.75E-10 |
| FEZF1_HUMAN.H11MO.0.C | 0.0590196 | 1.083391 | 1.16568 | 1.123704 | 2.81E-10 |
| PO5F1_HUMAN.H11MO.1.A | 0.04510565 | 1.095288 | 1.190992 | 1.142032 | 3.01E-10 |
| TEAD2_HUMAN.H11MO.0.D | 0.07421282 | 1.074098 | 1.146816 | 1.109819 | 3.10E-10 |
| NFIL3_HUMAN.H11MO.0.D | 0.02980541 | 1.118048 | 1.239977 | 1.177243 | 3.30E-10 |
| SOX17_HUMAN.H11MO.0.C | 0.04479985 | 1.09426 | 1.190183 | 1.141106 | 4.23E-10 |
| NFAC2_HUMAN.H11MO.0.B | 0.04145133 | 1.098138 | 1.198377 | 1.147038 | 4.44E-10 |
| GRHL2_HUMAN.H11MO.0.A | 0.07028327 | 1.074501 | 1.149229 | 1.111178 | 5.25E-10 |
| HMGA2_HUMAN.H11MO.0.D | 0.02425512 | 1.129248 | 1.267125 | 1.195962 | 5.57E-10 |
| PO4F3_HUMAN.H11MO.0.D | 0.03893867 | 1.099546 | 1.203209 | 1.150092 | 6.67E-10 |
| VENTX_HUMAN.H11MO.0.D | 0.02953019 | 1.114559 | 1.236614 | 1.173815 | 8.02E-10 |
| PO4F1_HUMAN.H11MO.0.D | 0.0379652 | 1.099949 | 1.205027 | 1.151167 | 8.38E-10 |
| ZFHX3_HUMAN.H11MO.0.D | 0.03051894 | 1.111823 | 1.231333 | 1.169868 | 9.38E-10 |
| FOXO3_HUMAN.H11MO.0.B | 0.05700132 | 1.080519 | 1.163995 | 1.121365 | 1.08E-09 |
| RAX2_HUMAN.H11MO.0.D | 0.02441821 | 1.1252 | 1.261921 | 1.191357 | 1.09E-09 |
| GFI1_HUMAN.H11MO.0.C | 0.05995739 | 1.078051 | 1.159197 | 1.117811 | 1.21E-09 |
| MSX1_HUMAN.H11MO.0.D | 0.0287402 | 1.113394 | 1.237026 | 1.173383 | 1.38E-09 |
| DUXA_HUMAN.H11MO.0.D | 0.02270063 | 1.128152 | 1.270815 | 1.197097 | 1.50E-09 |
| STA5A_HUMAN.H11MO.0.A | 0.06126724 | 1.076069 | 1.156174 | 1.115342 | 1.69E-09 |
| HNF1B_HUMAN.H11MO.0.A | 0.03203776 | 1.105781 | 1.221346 | 1.161961 | 1.87E-09 |
| ZN264_HUMAN.H11MO.0.C | 0.03766959 | 1.096527 | 1.201651 | 1.147726 | 2.11E-09 |
| PHX2A_HUMAN.H11MO.0.D | 0.02534071 | 1.118882 | 1.251875 | 1.183279 | 2.22E-09 |
| STA5B_HUMAN.H11MO.0.A | 0.05799007 | 1.076615 | 1.158966 | 1.116948 | 2.77E-09 |
| ONEC3_HUMAN.H11MO.0.D | 0.03670632 | 1.096306 | 1.202836 | 1.148178 | 3.08E-09 |
| LHX9_HUMAN.H11MO.0.D | 0.0269054 | 1.113507 | 1.2415 | 1.175563 | 3.14E-09 |
| HXB3_HUMAN.H11MO.0.D | 0.02907658 | 1.108533 | 1.230603 | 1.167815 | 3.27E-09 |
| ZN394_HUMAN.H11MO.1.D | 0.05199127 | 1.079394 | 1.166675 | 1.122099 | 4.22E-09 |
| ZN582_HUMAN.H11MO.0.C | 0.05020744 | 1.080555 | 1.169474 | 1.12403 | 4.73E-09 |
| VAX1_HUMAN.H11MO.0.D | 0.02773106 | 1.108432 | 1.2336 | 1.169163 | 5.87E-09 |
| SOX9_HUMAN.H11MO.1.B | 0.03890299 | 1.088544 | 1.190861 | 1.138447 | 9.70E-09 |
| ESR2_HUMAN.H11MO.1.A | 0.06772474 | 1.066942 | 1.142311 | 1.103925 | 9.75E-09 |
| DRGX_HUMAN.H11MO.0.D | 0.02589625 | 1.10957 | 1.239505 | 1.172515 | 9.79E-09 |
| GFI1B_HUMAN.H11MO.0.A | 0.06294405 | 1.069163 | 1.147563 | 1.10759 | 1.04E-08 |
| PBX2_HUMAN.H11MO.0.C | 0.02373526 | 1.113928 | 1.250807 | 1.180146 | 1.16E-08 |
| MYNN_HUMAN.H11MO.0.D | 0.03514673 | 1.092278 | 1.20063 | 1.145035 | 1.28E-08 |
| SHOX_HUMAN.H11MO.0.D | 0.02670153 | 1.106606 | 1.234011 | 1.168369 | 1.29E-08 |
| PO2F2_HUMAN.H11MO.0.A | 0.04121179 | 1.084873 | 1.18373 | 1.13312 | 1.31E-08 |
| PBX1_HUMAN.H11MO.1.C | 0.01843471 | 1.129413 | 1.289285 | 1.206384 | 1.42E-08 |
| NR2F6_HUMAN.H11MO.0.D | 0.06587974 | 1.066488 | 1.142888 | 1.103943 | 1.49E-08 |
| ESX1_HUMAN.H11MO.0.D | 0.02563122 | 1.107936 | 1.238339 | 1.171122 | 1.57E-08 |
| DUX4_HUMAN.H11MO.0.A | 0.0227414 | 1.1147 | 1.254845 | 1.182452 | 1.57E-08 |
| FOXP2_HUMAN.H11MO.0.C | 0.05257739 | 1.073848 | 1.160049 | 1.11604 | 1.71E-08 |
| PO6F1_HUMAN.H11MO.0.D | 0.02819995 | 1.101994 | 1.225019 | 1.161675 | 1.72E-08 |
| MGAP_HUMAN.H11MO.0.D | 0.04930532 | 1.075949 | 1.165193 | 1.11958 | 1.94E-08 |
| HXA1_HUMAN.H11MO.0.C | 0.0391884 | 1.084266 | 1.185707 | 1.133719 | 2.56E-08 |
| SHOX2_HUMAN.H11MO.0.D | 0.02614599 | 1.103535 | 1.231858 | 1.165749 | 2.77E-08 |
| PHX2B_HUMAN.H11MO.0.D | 0.02513175 | 1.105408 | 1.236808 | 1.169025 | 2.99E-08 |
| HNF1B_HUMAN.H11MO.1.A | 0.02677288 | 1.100526 | 1.226744 | 1.161732 | 3.74E-08 |
| ZN502_HUMAN.H11MO.0.C | 0.04678246 | 1.074133 | 1.165594 | 1.118834 | 5.20E-08 |
| GBX2_HUMAN.H11MO.0.D | 0.02605425 | 1.10017 | 1.2282 | 1.162209 | 5.29E-08 |
| PITX3_HUMAN.H11MO.0.D | 0.02973406 | 1.092212 | 1.210428 | 1.149629 | 6.68E-08 |
| PRRX1_HUMAN.H11MO.0.D | 0.02851595 | 1.094172 | 1.215319 | 1.152973 | 6.81E-08 |
| CDX2_HUMAN.H11MO.0.A | 0.0351926 | 1.083817 | 1.190955 | 1.135981 | 7.57E-08 |
| CEBPB_HUMAN.H11MO.0.A | 0.03019276 | 1.089906 | 1.20684 | 1.146689 | 9.03E-08 |
| CEBPA_HUMAN.H11MO.0.A | 0.03301632 | 1.085693 | 1.196669 | 1.139664 | 9.74E-08 |
| PAX3_HUMAN.H11MO.0.D | 0.02075879 | 1.108918 | 1.255023 | 1.17943 | 1.04E-07 |
| HME1_HUMAN.H11MO.0.D | 0.02533562 | 1.0947 | 1.223767 | 1.157236 | 1.87E-07 |
| GATA4_HUMAN.H11MO.0.A | 0.04053903 | 1.073508 | 1.171797 | 1.121448 | 2.10E-07 |
| NR1H2_HUMAN.H11MO.0.D | 0.04547262 | 1.067558 | 1.15957 | 1.112515 | 3.16E-07 |
| GATA1_HUMAN.H11MO.1.A | 0.04594151 | 1.066315 | 1.157711 | 1.110972 | 4.03E-07 |
| NFAC1_HUMAN.H11MO.1.B | 0.05326035 | 1.061302 | 1.145592 | 1.102533 | 4.33E-07 |
| BARX2_HUMAN.H11MO.0.D | 0.02600328 | 1.088101 | 1.214313 | 1.149274 | 4.48E-07 |
| BSH_HUMAN.H11MO.0.D | 0.0270583 | 1.086363 | 1.209698 | 1.146193 | 4.61E-07 |
| GATA2_HUMAN.H11MO.1.A | 0.04960603 | 1.062407 | 1.149903 | 1.105223 | 5.41E-07 |
| HXC11_HUMAN.H11MO.0.D | 0.03744534 | 1.07204 | 1.174259 | 1.12186 | 5.50E-07 |
| OTX1_HUMAN.H11MO.0.D | 0.03714463 | 1.07211 | 1.174755 | 1.122137 | 5.59E-07 |
| BARH1_HUMAN.H11MO.0.D | 0.03051894 | 1.07969 | 1.194441 | 1.135437 | 5.76E-07 |
| VSX1_HUMAN.H11MO.0.D | 0.02683404 | 1.08345 | 1.206874 | 1.143299 | 7.79E-07 |
| CUX2_HUMAN.H11MO.0.D | 0.02432138 | 1.087075 | 1.217677 | 1.150326 | 9.19E-07 |
| NFAC4_HUMAN.H11MO.0.C | 0.04409651 | 1.062802 | 1.155729 | 1.108196 | 1.21E-06 |
| PAX7_HUMAN.H11MO.0.D | 0.01849587 | 1.096428 | 1.249522 | 1.170196 | 1.64E-06 |
| CEBPD_HUMAN.H11MO.0.C | 0.03985097 | 1.063848 | 1.16177 | 1.111629 | 1.97E-06 |
| BARH2_HUMAN.H11MO.0.D | 0.03011631 | 1.072984 | 1.187631 | 1.128672 | 2.15E-06 |
| SOX18_HUMAN.H11MO.0.D | 0.0433422 | 1.060212 | 1.153663 | 1.105852 | 2.31E-06 |
| ISX_HUMAN.H11MO.0.D | 0.02419396 | 1.080568 | 1.210436 | 1.143441 | 2.68E-06 |
| HXB1_HUMAN.H11MO.0.D | 0.03767979 | 1.063903 | 1.164727 | 1.113049 | 2.80E-06 |
| NKX28_HUMAN.H11MO.0.C | 0.02937729 | 1.072243 | 1.188276 | 1.128595 | 2.84E-06 |
| HXA9_HUMAN.H11MO.0.B | 0.03206834 | 1.067787 | 1.177968 | 1.121365 | 3.65E-06 |
| PDX1_HUMAN.H11MO.0.A | 0.02471892 | 1.077114 | 1.204975 | 1.13904 | 3.94E-06 |
| HMX2_HUMAN.H11MO.0.D | 0.02455073 | 1.075173 | 1.203183 | 1.137168 | 5.47E-06 |
| DLX2_HUMAN.H11MO.0.D | 0.02688501 | 1.070564 | 1.191826 | 1.129349 | 6.65E-06 |
| MEOX2_HUMAN.H11MO.0.D | 0.03091139 | 1.064795 | 1.176654 | 1.119171 | 7.58E-06 |
| TF7L1_HUMAN.H11MO.0.B | 0.04083463 | 1.054947 | 1.150602 | 1.101618 | 1.01E-05 |
| PAX4_HUMAN.H11MO.0.D | 0.02792473 | 1.066247 | 1.184471 | 1.123619 | 1.05E-05 |
| OTX2_HUMAN.H11MO.0.A | 0.03721599 | 1.056998 | 1.157593 | 1.106036 | 1.12E-05 |
| HXB7_HUMAN.H11MO.0.C | 0.02451505 | 1.06855 | 1.195549 | 1.130063 | 1.53E-05 |
| HSFY1_HUMAN.H11MO.0.D | 0.03397959 | 1.05749 | 1.16302 | 1.108855 | 1.65E-05 |
| TBP_HUMAN.H11MO.0.A | 0.03882654 | 1.053346 | 1.151294 | 1.101111 | 1.78E-05 |
| MEIS1_HUMAN.H11MO.0.A | 0.02959135 | 1.059616 | 1.173307 | 1.114855 | 2.34E-05 |
| HXD8_HUMAN.H11MO.0.D | 0.0307381 | 1.057105 | 1.168193 | 1.111113 | 2.97E-05 |
| HXB8_HUMAN.H11MO.0.C | 0.02148252 | 1.067853 | 1.204001 | 1.133646 | 3.25E-05 |
| HNF6_HUMAN.H11MO.0.B | 0.02088621 | 1.06857 | 1.206912 | 1.135388 | 3.49E-05 |
| LHX3_HUMAN.H11MO.0.C | 0.02763422 | 1.059067 | 1.176838 | 1.116226 | 3.51E-05 |
| HXD9_HUMAN.H11MO.0.D | 0.01300674 | 1.083388 | 1.265975 | 1.170702 | 5.62E-05 |
| HXD4_HUMAN.H11MO.0.D | 0.01698725 | 1.070831 | 1.225922 | 1.145433 | 6.69E-05 |
| PO4F2_HUMAN.H11MO.0.D | 0.02977483 | 1.052793 | 1.165122 | 1.107368 | 6.70E-05 |
| PDX1_HUMAN.H11MO.1.A | 0.02654863 | 1.053831 | 1.173329 | 1.111779 | 9.19E-05 |
| HXA7_HUMAN.H11MO.0.D | 0.0215233 | 1.055884 | 1.189811 | 1.12061 | 1.50E-04 |
| NOBOX_HUMAN.H11MO.0.C | 0.02104421 | 1.054498 | 1.189811 | 1.119871 | 1.93E-04 |
| LHX4_HUMAN.H11MO.0.D | 0.02449976 | 1.04647 | 1.169897 | 1.106275 | 3.25E-04 |
| HXB6_HUMAN.H11MO.0.D | 0.02409203 | 1.042785 | 1.166747 | 1.102818 | 5.50E-04 |
| HMX3_HUMAN.H11MO.0.D | 0.02280257 | 1.043833 | 1.17162 | 1.105676 | 5.61E-04 |

OR represent odds ratio.

## Supplementary materials

**1.3.1 Supplementary R code.** R code for calculating methylation age

R code for calculating methylation age:

<https://github.com/Markzheng5/DNAmage.git>

R code for ssGSEA:

<https://github.com/Markzheng5/ssGSEA.git>
